# Innovation Process and Industrial System of US Food and Drug Administration–Approved Software as a Medical Device: Review and Content Analysis

**DOI:** 10.2196/47505

**Published:** 2023-11-24

**Authors:** Jiakan Yu, Jiajie Zhang, Shintaro Sengoku

**Affiliations:** 1 Department of Technology and Innovation Management School of Environment and Society Tokyo Institute of Technology Tokyo Japan; 2 Graduate School of Interdisciplinary Information Studies The University of Tokyo Tokyo Japan; 3 Department of Innovation Science School of Environment and Society Tokyo Institute of Technology Tokyo Japan

**Keywords:** digital health, digital therapeutics, software as a medical device, innovation process, artificial intelligence

## Abstract

**Background:**

There has been a surge in academic and business interest in software as a medical device (SaMD). SaMD enables medical professionals to streamline existing medical practices and make innovative medical processes such as digital therapeutics a reality. Furthermore, SaMD is a billion-dollar market. However, SaMD is not clearly understood as a technological change and emerging industry.

**Objective:**

This study aims to review the landscape of SaMD in response to increasing interest in SaMD within health systems and regulation. The objectives of the study are to (1) clarify the innovation process of SaMD, (2) identify the prevailing typology of such innovation, and (3) elucidate the underlying mechanisms driving the SaMD innovation process.

**Methods:**

We collected product information on 581 US Food and Drug Administration–approved SaMDs from the OpenFDA website and 268 company profiles of the corresponding manufacturers from Crunchbase, Bloomberg, PichBook.com, and other company websites. In addition to assessing the metadata of SaMD, we used correspondence and business process analysis to assess the distribution of intended use and how SaMDs interact with other devices in the medical process.

**Results:**

The current SaMD industry is highly concentrated in medical image processing and radiological analysis. Incumbents in the medical device industry currently lead the market and focus on incremental innovation, whereas new entrants, particularly startups, produce more disruptive innovation. We found that hardware medical device functions as a complementary asset for SaMD, whereas how SaMD interacts with the complementary asset differs according to its intended use. Based on these findings, we propose a regime map that illustrates the SaMD innovation process.

**Conclusions:**

SaMD, as an industry, is nascent and dominated by incremental innovation. The innovation process of the present SaMD industry is shaped by data accessibility, which is key to building disruptive innovation.

## Introduction

### Background of Software as a Medical Device

The widespread adoption of digital technology has led to the emergence of digital health, including mobile health (mHealth) and telemedicine, and has transformed the way software is used in the health care industry. Software developed for medical purposes is no longer limited to serving as an embedded function in hardware or stand-alone devices; rather, it has become a crucial aspect in advancing public health. To mitigate the potential risks associated with this shift, the International Medical Device Regulators Forum (IMDRF) has defined software as a medical device (SaMD). SaMD refers to software that is intended to be used for one or more medical purposes and is capable of performing these purposes without being part of a hardware medical device [1,2]. In general, traditional medical devices, such as magnetic resonance imaging (MRI) or endoscopy, assume that the end user manipulates the device to deliver medical procedures. As the internet of things, especially wearable devices, has gained popularity, SaMD can engage patients as end users in the delivery of medical services. In other words, SaMD has the potential to realize patient-centric health care by promoting behavioral changes in patients, conducting real-time patient monitoring, and improving patient-doctor communication. This helps address the challenges caused by a rapidly aging society [3]. With the shift in population distribution toward older age, endogenous diseases, also known as lifestyle-related or aging-related diseases, have increased. Unlike exogenous diseases, such as COVID-19, endogenous diseases are caused by multiple factors, which means that a complete cure is nearly impossible. The health care system needs to adapt to changes and deliver early detection and progression control of endogenous diseases [4]. The use of digital technology, such as SaMD, facilitates patient-centered preventive and therapeutic interventions. It also enhances the capacity for an accurate diagnosis and improves health care delivery [5-9]. This emerging industry generates billions of dollars in revenue. As of 2019, the global market size of SaMD has reached US $18.5 billion and is projected to grow rapidly at an annual rate of 21.9% by 2027 [10].

### Issues and Challenges for SaMD

The SaMD has gained popularity in academia over the past decade. Previous studies have focused on 2 main topics: addressing the technical challenges in delivery and revealing how the existing regulatory framework should adapt to technological changes. Studies regarding technological implementation primarily concentrate on engineering problems, such as cybersecurity and reliability of internet of things devices, in delivering applicable digital health functions [11-14]. The studies discussed herein aim to address the technical challenges that arise when developing digital health or SaMD, based on specific cases or applications, from an engineering perspective. Other studies have focused on the current regulatory frameworks and regulatory oversight [15-18]. Most studies focus on medical devices embedded with artificial intelligence (AI) and machine learning (ML) algorithms and propose that regulatory requirements need to be strengthened to ensure effective regulation [15,16,19]. Several studies have conducted international comparisons of regulatory frameworks to identify the focus of regulatory oversight in various countries [17,18]. SaMD is a part of the research subject, which includes hardware medical devices. Although existing literature have provided valuable insights into the challenges posed by SaMD, they have certain limitations. First, they did not provide a holistic view of SaMD. The empirical data set in support of previous studies is limited to pilot data or specific topics, such as AI- and ML-based medical devices [15-17,19]. Therefore, there is limited knowledge available about the overall landscape of the SaMD industry, which includes both AI- and ML-based SaMDs and those that are not. Although AI- and ML-based SaMDs may be heterogeneous compared with others, results based on a confined universe do not reveal a complete map of the SaMD industry. Furthermore, previous studies have primarily focused on the challenges posed by regulatory frameworks [15,17,18]. Few studies have addressed the SaMD dynamics from the perspective of innovation management. One reason for this is that regulators in countries, such as the European Union, Japan, and the United States have not provided a pathway to identify regulator-approved SaMDs in database of medical device registration. The unavailability of data poses a challenge for innovation management researchers to explore the SaMD universe [15,17-19].

### Aim and Objectives

Based on the considerations outlined above, this study addresses the limitations of the present research on SaMDs by exploring the product features, manufacturers, and medical processes of all US Food and Drug Administration (FDA)–approved SaMDs over the past decade. Using this data set, this study aims to achieve the following objectives: (1) clarify the market landscape and formation process of SaMD as an industry; (2) identify the role of SaMD in the entire medical process; and (3) examine the underlying mechanisms driving SaMD innovation. The empirical findings are presented in a regime map illustrating how SaMD has emerged as a part of the medical device industry and will continue to shape it in the future.

## Methods

### The Data Set

**Figure 1:**
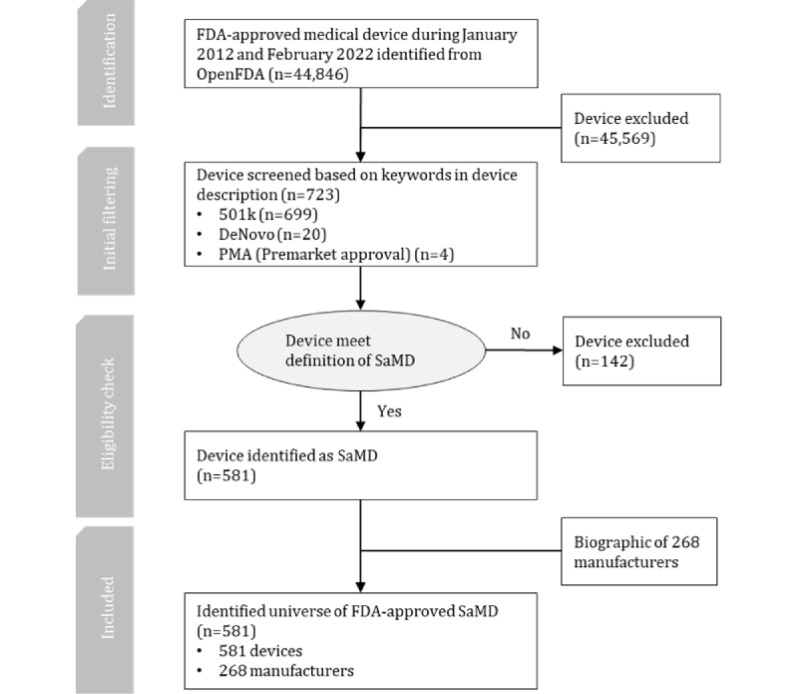
Data extraction and aggregation approach. FDA: US Food and Drug Administration; PMA: premarket approval; SaMD: software as a medical device.

This study is based on SaMD approved by the FDA, the sole authority that issues approvals for commercially marketed medical devices in the United States. Information on all approved medical devices is published in OpenFDA, a database that provides device descriptions, indications for use, and decision summaries of all FDA-approved medical devices [20-22]. All devices are categorized into 3 pathways: premarket approval, 501(k) approval, and De Novo premarket review. However, no regulatory pathway recognizes SaMDs. We referred to the methods applied by Wu et al [15] to extract device information from OpenFDA and identify devices that met the definition of SaMD using keyword filtering. First, we downloaded product information for 44,846 FDA-approved medical devices in PDF and HTML format. To obtain a complete landscape of FDA-approved SaMDs, this study extracted product information for all FDA-approved medical devices between January 2012 and February 2022 and filtered all devices that met the definition of SaMD. We chose January 2012 as the starting point of the search because the IMDRF began discussing the definition of SaMD in 2012 [1]. With the product information included in the device descriptions, we filtered 723 devices that are counted as SaMD using keywords and extracted the corresponding information to create an initial SaMD data set (Figure 1). The keywords used to identify SaMD included: “Software as Medical Device,” “Software as a Medical Device,” “Standalone software,” “Software only,” “Software package,” and “Software device.” To be considered as SaMD, devices need to function as stand-alone software that fulfills medical functions. Devices are excluded if they functioned only as part of the hardware or required hardware to fulfill their intended use. Finally, we checked the descriptions of all selected devices and identified 581 devices eligible for SaMD. To gain a comprehensive understanding of the selected devices, we extracted the company name of 268 manufacturers as complementary information using the applicant names shown on the Open FDA website. As no comprehensive database encompassing profiles of all 268 manufacturers existed, we conducted manual searches on Crunchbase, Bloomberg, PitchBook, and the respective company websites. We systematically verified each company on all those websites to procure comprehensive company profiles, such as establishment date, business scale, number of employees, etc. Crunchbase is a reputable platform known as comprehensive database of startups [23]. Bloomberg is a renowned global financial information provider who provides company profile for most middle and large enterprises and institutions worldwide [24]. PitchBook specializes in private market data and is widely recognized for its in-depth coverage of private equity, emerging businesses, and startups [25]. We took precautions to cross-reference data from these websites, ensuring data accuracy. The entire data set represented a compilation of product information for 581 FDA-approved SaMDs and biographical information for 268 manufacturers.

### Descriptive Statistics

Based on the data set extracted from the OpenFDA website, we conducted descriptive statistics to explore the SaMD industrial system in terms of its development process, innovator biography, and product specialty. The numbers of SaMDs developed over the past decade are aggregated to specify the development process. Additionally, the number of SaMDs are aggregated by manufacturers’ biographies to determine the distribution of devices by nationality, company size, and original industry of manufacturers.

### Correspondence Analysis

To understand the structure of industrial systems, we conducted a correspondence analysis considering the number of SaMD approvals per manufacturer and medical specialty to analyze the structure of the industrial system from the perspective of specialty concentration. The contingency table is coded with the number of SaMDs in the rows for 13 medical specialties and columns for 268 manufacturers. Initially, to classify manufacturers, we conducted agglomerative hierarchical clustering using the Ward method and calculated the pairwise distance using the Euclidean distance measure. We evaluated clustering using the indices proposed by Calinski and Harabasz [26], Hartigan [27], and Krzanowski and Lai [28], and identified 5 clusters of SaMD manufacturers (Table S2 and Figure S5 in Multimedia Appendix 1). Second, we created a contingency table coded with the number of SaMD in rows for 13 medical specialties and columns for the 5 identified clusters of manufacturers. Using a contingency table, we conducted a correspondence analysis to investigate the relationship between SaMD manufacturers and medical specialties (Tables S3, S4, S6, and S7 in Multimedia Appendix 1). We selected Dim1 and Dim2, the first and second principal components of the correspondence analysis, to present the result. Because Dim1 and Dim2 presents the highest cumulative percentage of variance (97.33%) which means most information contained in the source data is aggregated in 1 plane.

### Business Process Analysis

To differentiate between SaMDs, we need to identify not only the function delivered by each SaMD but also the contextual functions provided by other devices that enable the SaMD to fulfill its intended use. Taking a holistic view of medical practice helps us understand the role of SaMD in delivering its intended use. To identify the underlying features of the entire process in which SaMD is engaged, we conducted a business process analysis using a SIPOC (suppliers, inputs, process, outputs, and customers) diagram, a visual rendering of Dr W Edwards Deming’s system model, which is commonly used in total quality management [29-31]. Supplier supplies the inputs necessary to complete the entire process outlined by the system. The input is everything a system requires to complete all processes. The process involves the actions necessary to produce the result intended by the system. The output is the final result of the system. The customer receives the results produced by the system. SIPOC diagram enables visualization of the pattern of a system that consists of several processes from a holistic view, instead of treating the engaged processes as individual pieces [32].

**Figure 2:**
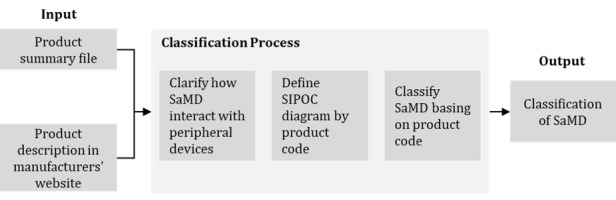
Classification process of software as a medical device (SaMD). SIPOC: suppliers, inputs, process, outputs, and customers.

Following the process shown in Figure 2, a total of 581 SaMDs are classified according to the pattern of the SIPOC diagram. First, we read the summary file of each SaMD and checked the device description on the manufacturer’s website to specify how the SaMD interacts with the complementary devices. The summary file contains a detailed description of the intended use of each device, whereas the product description on the manufacturer’s website explains how the complementary device is engaged. After reviewing the 581 files, we found that SaMD with the same product code showed a similar method of delivering the intended use. The FDA classifies medical devices by product codes, a coding system developed by the Center for Devices and Radiological Health, to categorize medical devices with similar intended use, medical specialty, and product characteristics. Based on this finding, we defined the SIPOC diagram using the product code based on the SaMD in that product code. Finally, we identify 3 business process patterns.

## Results

### Overview of SaMD as an Industry

In 2012, the FDA approved the first device to meet the IMDRF definition of SaMD. Since then, the cumulative number of SaMDs approved by the FDA has increased from 1 device in 2012 to 581 in 2021. Between 2012 and 2022, the number of FDA-approved SaMDs increased at a compound annual growth rate of 202.7%. Additionally, AI- or ML-based SaMD is first approved by the FDA in 2016 and is subsequently increased to 41 in 2020 and 37 in 2021. Cumulatively, AI- and ML-based SaMDs account for 22% of all FDA-approved SaMDs. The surge in AI- and ML-based SaMDs can be attributed to the “third AI boom” in health care (Multimedia Appendix 2).

In terms of countries, the United States (n=262, 45%) launched the most SaMD devices during the past decade, followed by Germany (n=71, 12%), South Korea (n=32, 5.5%), and the Netherlands (n=27, 4.6%). The United States is undoubtedly the leader in developing new SaMDs. Corporations of United States nationality have approved 262 SaMD devices by the FDA in the past decade. This number covers 45% (262/581) of the total 581 FDA-approved SaMD devices and 191 more than the 71 devices from Germany, which is the country with the second most approved SaMD devices. Another difference between the 2 countries is that 42 devices are from public companies in United States, whereas the remaining 220 devices are from unlisted companies, startups, or even seed companies. In Germany, 51 devices are from public companies, such as Siemens and Carl Zeiss Meditec. The startup ecosystem in the United States merits recognition for bringing innovations to the market. Similar to what Onodera saw in mHealth [33], our observation supports her anticipation that the United States, sustained by its industrial and entrepreneurial ecosystem, will be the hub for commercializing innovation in digital health [34]. In terms of manufacturers, large companies like Siemens, General Electric Company (GE), and Philips launched the most SaMD devices (n=237, 40.8%), followed by small and micro companies (n=215, 37%), most of which are established after 2012. We found that incumbents develop SaMD devices as peripheral products to strengthen their competitive advantage in the existing medical business, as is the case with Siemens, GE, and Philips in their medical imaging business. In terms of product specialty, the use of SaMD devices for radiology (n=452, 78%) is the most common, followed by cardiovascular (n=54, 9%), neurology (n=26, 4%), ophthalmology (n=15, 3%), and dentistry (n=10, 2%). We investigated the regulatory descriptions of the medical specialties and found that most SaMD devices categorized in radiology specialize in image processing and analysis (n=378). In terms of industrial classification, medical equipment, devices, and software manufacturers led the market with 385 devices (66%; Figures S1-S4 in Multimedia Appendix 1). On the other hand, the market witnessed increasing mergers and acquisitions in the past 3 years (Table S1 in Multimedia Appendix 1). Since 2012, 43 of the 268 identified manufacturers have been acquired by or merged with another corporate in the industry. In particular, 21 of the 43 identified deals occurred over the past 3 years.

### Market Characteristics by Manufacturers and Usages

**Figure 3:**
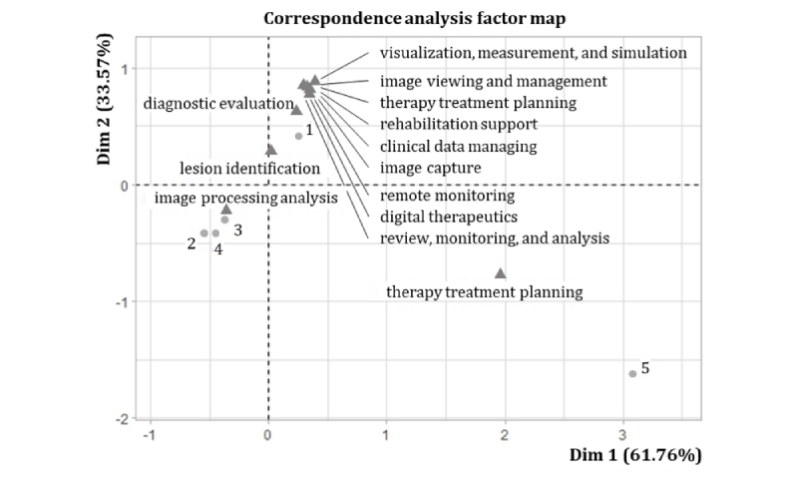
Correspondence analysis clustered by manufacturers and usages. The correspondence analysis is based on principal component analysis, Dim1 and Dim2 mean the first and second principal components, respectively. Circle spots represent the 5 groups of manufacturers and triangles represent medical specialty of software as a medical device.

The result of the correspondence analysis is shown in Figure 3, in which circle spots represent the 5 clusters of manufacturers and triangles represent medical specialty of SaMD. To interpret the correspondence analysis, the first step is to evaluate whether there is a significant dependency between the manufacturing cluster and medical specialties. A high chi-square statistic indicates a strong link between the row and column variables. In our analysis, this association is highly significant (*χ*^2^_48_=378.53; *P*<.001). Contribution and squared correlation for Dim1 and Dim2 indicate that “therapy treatment planning” and “therapy treatment planning” are well explained by Dim1, while the other medical specialties are well explained by Dim2. With regard to the relations between clusters of manufacturers and medical specialties, we found that cluster 1 locates close to diversified medical specialties; clusters 2, 3, and 4 locates close to “image processing analysis”; and cluster 5 locates close to “therapy treatment planning.” This indicates the concentration of medical specialties among the 5 identified clusters of manufacturers.

Cluster 1 includes 300 devices marketed by 232 manufacturers, the majority of which are small and micro companies. These manufacturers focus on diversified medical specialties and market only 1 device per manufacturer, on average. In contrast to clusters 2, 3, 4, and 5, cluster 1 is fragmented in terms of medical specialty and number of devices. Some revolutionary applications such as digital therapeutics, rehabilitation support, and remote monitoring, which realize patient-centric health care, are categorized in cluster 1.

Clusters 2, 3, and 4 contained 265 devices marketed by 53 manufacturers, most of which are incumbent and large companies. These manufacturers focus on image processing analysis and market 5 devices per manufacturer, on average. Specifically, the incumbents dominated the market. Siemens, GE, Philips, Canon, and Fujifilm developed 93 SaMDs for image processing and analysis. Cluster 2 represents 54 companies that launched 142 SaMDs, 101 of which are launched by new entrants with a history of less than 20 years.

Cluster 5 presents 16 devices marketed by 1 manufacturer, Varian Medical Systems, acquired by Siemens in 2021. Varian is a giant incumbent in radiation oncology treatment with a history of more than 50 years. All SaMDs in this group are designed for the LINAC (linear accelerator) or MRI device of Varian for therapy planning. Additionally, Varian is acquired by Siemens in 2021.

### Product Classification and Investigation

To provide a comprehensive understanding of SaMD in the context of medical diagnosis and treatment, we have categorized the 581 SaMD products based on their functions and their application in different medical specialties. This categorization resulted in the identification of 3 patterns that illustrate the variances in how SaMD interacts with other hardware devices (Table S5 in Multimedia Appendix 1).

A total of 82 SaMD fall under pattern 1. In this category, SaMD converts the analog data collected by hardware into a user-friendly format for visualization. Among them, 4 SaMDs are AI- and ML-based. These SaMDs are primarily used in radiology (n=51), followed by cardiovascular (n=12), anesthesiology (n=6), and neurology (n=5). They are installed as stand-alone software in off-the-shelf workstations to enhance the usability of the data but do not provide new insights from the data through analysis or modeling. These SaMDs are similar to off-the-shelf software, such as Windows Office, and are designed for simple processing, data visualization, and management. Generally, SaMDs in pattern 1 refer to a data set collected by hardware medical devices through a standard off-the-shelf workstation or PC. For example, Carestream’s bone-suppression software creates a companion image and suppresses the appearance of the bone to improve the visualization of soft tissue using data collected in projectional radiography. SonomedEscalon’s AXIS Image Management System is a web-based system tailored for ophthalmic image management, facilitating the archival, retrieval, and assessment of ophthalmic images, videos, and reports generated by ophthalmic imaging devices.

A total of 461 SaMDs fall under pattern 2. SaMDs in this category processes data collected by hardware to detect features through modeling. Overall, 124 out of these SaMDs are AI- and ML-based SaMDs. These SaMDs are primarily used in radiology (n=415), followed by cardiovascular (n=17), neurology (n=11), and dentistry (n=10). These SaMDs in pattern 2 are installed as a stand-alone software in workstations to enhance data usability through detecting features. They are similar to off-the-shelf software, such as STATA, which is a general-purpose statistical software package conducting data analysis or interpretation. Intended use is more complex than SaMDs in pattern 1. In pattern 2, SaMDs require a large volume of data sets to develop, verify, and improve its algorithms. The results of data modeling are used by medical professionals to assist with existing medical processes, including lesion identification, diagnostic decisions, and 3D simulation. Most SaMDs in pattern 2 are independent of hardware medical devices and read data through a standard off-the-shelf workstation. However, 80 SaMDs are exclusively postprocessing applications designed for specific medical devices. For example, Siemens’ Syngo Dual Energy is an SaMD designed to operate with Siemens dual-source computer tomography scanners.

Only 38 SaMDs fall under pattern 3. SaMD uses data collected by hardware to detect features through modeling. The intended uses, such as digital therapeutics and remote monitoring, are new to the existing health care systems. Only 1 device from Apple Inc is AI- and ML-based SaMD. These SaMDs are primarily used in the cardiovascular (n=21), neurology (n=10), and other nonclassified medical specialties (n=3). Similar to the SaMD in pattern 2, 7 out of the 38 devices are installed as stand-alone software in workstations to enhance data by detecting features, while the intended use is for new medical processes. A total of 31 of the 38 devices are integrated with data-collection hardware in a single product. Generally, the hardware in pattern 3 is customized devices or mobile phones that functions as both a data collector and an interface with the user. For the 31 devices, the modeling results are used by the hardware to interact with the user, who is a trained medical professional or patient. For example, Luminopia One is a digital therapy device that incorporates dichoptic presentations on visual displays using SaMD, a therapeutic algorithm used to treat amblyopia or improve the visual acuity of patients with amblyopia. In the case of Luminopia, SaMD functions as a stand-alone component for postprocessing, whereas the hardware works as a data collector and user interface.

## Discussion

### Trend of SaMD Market Formation

The fast-growing number of FDA-approved SaMD is evidence of the increasing investment in digital health [35]. This growth is driven by both incumbents, who are already major players in the medical device industry, and new entrants, who bring expertise from software engineering to health care. The former group focuses on certain medical specialties, as the majority of the SaMDs they launch are peripheral products that strengthen their competitive advantage in their existing businesses. The latter group provides applications in diverse areas, such as digital therapeutics which are new to the existing health care system. In technology management, innovation is classified into 2 categories: incremental innovation, which improves the efficiency and cost-effectiveness of established, complex products or services, or disruptive innovation, which redefines and expands the boundaries of existing product or services [36]. In mHealth, disruptive innovation pertains to new products or practices into new markets or medical practice, whereas incremental innovation pertains to adaptation or enhancement of existing products within either existing or peripheral domain of existing markets [33]. Considering SaMD in terms of typologies of the novelty, we define SaMD as incremental innovation when it optimizes existing medical practices and as disruptive innovation when it enables new medical practice like digital therapeutics. In other words, our analysis shows that incumbents focus on incremental innovation, while new entrants focus on disruptive innovation.

Currently, most FDA-approved SaMDs are incremental innovations embedded in existing medical processes as tools to enhance the efficiency or accuracy of medical professionals. Notably, the industry is dominated by medical image processing. In recent years, with the sophistication of medical imaging equipment, large amounts of image data have accumulated in the medical field. The need for improved resolution and labor-saving methods for image interpretation is increasing. It is understandable that the application of SaMD, which exerts medical effects based on data, focuses on medical image processing [37,38]. Disruptive innovations, such as digital therapeutics and long-awaited revolutionary applications are still a minority in the market. Similar to what is seen in mHealth [33], the current SaMD industry concentrates on the existing market, where use cases and data storage are well developed. Onodera and Sengoku’s [33] observation of mHealth supported Christensen’s anticipation that IT can connect patients with medical professionals [39]. We also observed applications, such as real-time patient monitoring, in the current SaMD industry. However, the SaMD industry that Christensen anticipated is yet to be achieved.

Although incumbents and new entrants seem to have different focuses in developing SaMD, our data show that incumbents are extending their capabilities in SaMD through mergers and acquisitions. The increasing investment toward digital health signals that new medical technology will continue to grow [35]. For incumbents seeking new business or existing unicorn venture wanting to meet high-growth expectations, inorganic growth through mergers and acquisitions becomes necessary. For instance, Siemens acquired Varian, a listed company that provides solutions for radiotherapy. Siemens aims to address the growing need for personalized, data-driven diagnosis and precise cancer care [40]. The other acquisition happened to startup or ventures whose innovative technology is complementary to the existing business of incumbents. To strengthen its patient-monitoring business, Philips acquired full or part of the business of Cardiologs, TomTec Imaging Systems, and the IT business of Carestream. Nanox acquired Zebra Medical Vision, a deep learning medical imaging analytics company, for a US $200 million deal in 2021 [41]. Similar to our view toward the number of marketed SaMDs in the future, we estimate that incumbents and giant ventures will continue to acquire innovative startups to expand their technological capabilities.

The increasing number of mergers and acquisitions in SaMD signals that SaMD is an emerging blue ocean for medical device manufacturers, and a holistic market solution is needed to win. Our analytical results suggest that the increase in strategic patronage between incumbents that lack the in-house capability to bring their own SaMD solutions and new entrants bringing digital knowhow to the medical industry is driving this trend. The top 5 pharmaceutical companies invested US $270 million in SaMD initiatives between January 2019 and October 2021 [42]. Strategic partnership is the preferred way for pharmaceutical companies to bring their SaMD solutions to the market. A total of 73% of the SaMDs brought by pharmaceutical companies have strategic partners [42]. Similar to what happened in mHealth, pharmaceutical companies will actively engage in the digitalization process of health care [33,43,44].

SaMD, a promising subsector of digital health, has the potential to revolutionize health care, including monitoring, patient care, and therapies. Understanding SaMD from the perspective of innovation management helps investigate how to facilitate the emergence of innovation, which creates new markets and growth [39,45]. This insight into market formation is particularly valuable for business strategists because a new market usually requires a new business strategy, which is the key to success [46].

### Characteristics of SaMD Manufacturers

The current SaMD industry is highly concentrated in certain medical specialties such as medical image processing and radiology analysis. SaMD, as a kind of software, relies on input to deliver the designed function. The input data come from interactions with users or digital signals collected by the hardware. The mature method of collecting data and the accumulation of structured or semistructured image data have facilitated the concentrated product distribution of SaMD. In other words, the lack of data accumulation or data collection methods has impeded the emergence of SaMD in a diversified distribution by medical specialty.

Incumbents, especially giant players in the medical device industry, can build their own data while preferring to develop SaMD as a complementary product to their existing business. Most FDA-approved SaMDs developed by companies, such as Siemens and GE, concentrate on their MRI or computer tomography business. Philips provides SaMD for a central monitoring system, while still for its patient monitoring business. However, data are critical for applications that do not rely on existing medical processes. To develop applications such as digital therapeutics or remote monitoring, manufacturers must build their own data storage from scratch, which requires a huge investment, while it is small firms or ventures that drive disruptive innovation in SaMD.

A lack of data can impede the emergence of disruptive innovation in SaMD, as small firms and ventures usually cannot afford to build a database for a single product. Furthermore, SaMD requires continuous improvement, particularly in the evaluation and improvement of AI- and ML-based SaMD, which requires prospective studies. However, nowadays, all FDA-approved AI- and ML-based devices have only been evaluated by retrospective studies [15]. SaMD as an industry will not prosper if data accessibility remains difficult. One potential solution is to build an independent data infrastructure that provides open access to anonymized medical data. The initiator can be the government or a third-party authority such as an academic institution. Building a data infrastructure to provide data accessibility as a public good to potential innovators in health care can definitely benefit a country with the highest proportion of an aging population. No matter what kind of scheme it will be, we believe that data accessibility is a catalyst that drives the further development of SaMD.

### Regime of Innovation

**Figure 4:**
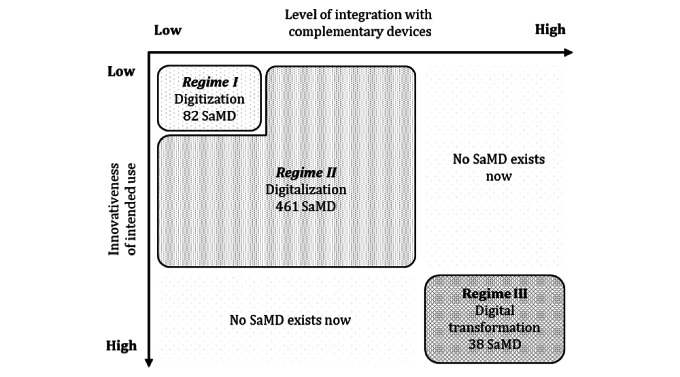
Regime map of innovation in software as a medical device (SaMD).

Our findings show that the less the intended use of the SaMD is relevant to the existing medical process, the higher the level of integration of the SaMD with complementary devices. Integration with complementary devices increases as intended use becomes more innovative. Based on our empirical findings, we propose a regime map to describe and explain SaMD’s innovation processes (Figure 4). The regime of innovation in SaMD is described by 2 key factors: the innovativeness of intended use and the level of integration with complementary devices. The innovativeness of intended use refers to the relevance of the SaMD to existing medical processes, whereas the level of integration with complementary devices indicates the extent to which the SaMD relies on complementary devices to deliver its intended use. We defined 3 regimes: digitization, digitalization, and digital transformation, corresponding to patterns 1, 2, and 3, as listed in Table 1.

**Table 1:** Classification and investigation of software as a medical device (SaMD) using suppliers, inputs, process, outputs, and customers diagram.

Pattern	Supplier	Input	Process	Output	Customer
Pattern 1 (82 SaMD)	Standard off-the-shelf workstation (PC)	Data collected by off-the-shelf hardware medical device	Data enhancement	Data visualization	Medical professionals
Pattern 2 (461 SaMD)	Standard off-the-shelf workstation (PC)	Data collected by off-the-shelf hardware medical device	Data enhancement and feature detection	Data visualization and clinical interpretation	Medical professionals
Pattern 3 (38 SaMD)	Customized hardware	Data collected by customized hardware	Data enhancement and feature detection	Clinical interpretation and user interaction	Patient and medical professionals

In regime I (digitization), SaMD conducts basic processing to streamline existing medical processes by changing the data format. Their intended use is so simple that data are not a critical resource for developing outstanding SaMDs. The data for these SaMDs are the processing targets stored in a workstation. SaMD is independent of the complementary device during the entire process. SaMD prospers from where needs exist and are loosely distributed. In regime II (digitalization), SaMD extends or enhances the capability of trained medical professionals to produce clinical interpretations. The intended use requires advanced analytics, for which data are critical, to develop an SaMD. Data for these SaMDs are not only processing targets stored in workstations, but also a resource to be outstanding. SaMD is concentrated on medical specialties with extensive data accumulation. In this regime, SaMD shows higher integration with the hardware because a certain number of SaMDs are designed exclusively for other hardware. In regime III (digital transformation), SaMD delivers a new medical process that does not exist in the current health care system and targets patients more than trained medical professionals. Similarly, the intended use requires advanced analytics; however, data accumulation is typically rare in this case. SaMD relies on a complementary device that collects data to deliver its intended use, whereas the complementary device requires input from the SaMD to deliver designed functions such as user interaction. The SaMD in regime III is dispersed in the medical specialty as all applications lack data accumulation.

Overall, SaMD prospered as an industry, with the majority of products concentrated in the d digitalization regime. Similar to mHealth [16], SaMD is dominated by incremental innovations, which streamline the existing medical practice. A total of 543 SaMD located in the digitization and digitalization categories are designed for existing medical processes. Only 38 SaMDs located in regime III are designed for disruptive innovation, such as digital therapeutics and patient behavior changes. The innovation process of SaMD so far is an expansion from existing medical specialty with sufficient data accumulation to new medical specialty need complementary hardware to build its own data accumulation. The more innovative the intended use of SaMD, the more necessary it is to integrate it with complementary hardware.

In addition, the distinction between regimes provides insights into market competition for SaMD [47,48]. Products in regimes I and II enjoy flexibility because of the lower interdependency of their components. This flexibility is an advantage for tangible goods in catching up with new technological changes, whereas for SaMD, it means that they can be easily replaced. For SaMD, which is designed for simple processing, embedding it into real-world medical practice is a way to outperform its competitors. This can be achieved through a user-friendly interface or unique functions required in medical practice. For SaMD designed for advanced analytics, enhancing the excellence of the algorithm is required, which can be achieved by involving real-world data sets in the development process. Manufacturers that can access high-quality real-world data outperform others. On the other hand, the products in regime III are characterized by a high level of integration with complementary devices. The excellence of SaMD in regime III relies on the combined performance achieved with the hardware in which SaMD is integrated. To outperform competitors, a manufacturer must build its hardware development capabilities, including user interface design, data collection, and product design.

### Limitations and Future Perspectives

First, there is no official pathway for filtering SaMDs from the FDA database. A filtering method other than keyword filtering can lead to new findings about the market, although we believe that this study is a good trial for innovation management studies of SaMD. Second, the 581 SaMDs and 268 manufacturers extracted from FDA databases, spanning the period from 2012 to 2021, represent the perspective of the United States, while naturally the method excludes SaMDs only register in other regulatory authority like the European Union, China, and Japan. We believe that the innovation of SaMD in other markets or countries may differ from what we saw in the United States. Consequently, it deserves further academic efforts to investigate the characteristics of SaMD in those markets or countries. Third, product architecture, that is, the composition of SaMD and complementary products and databases, has not been fully explored in this study, while this is a prerequisite for successful product development, especially in regime 3. Finally, the manner in which the proposed framework fits specific business cases of SaMD requires further investigation.

### Conclusions

SaMD is an industry with a biased structure that concentrates on medical specialties or applications where data accumulation and data collection methods are available. Incumbents have led the industry to date, as they have launched most SaMDs in the market. In contrast, new entrants or startups have diversified applications in the market and tend to initiate more disruptive innovations in diversified medical specialties. Regarding the mechanism that produces the market structure, we propose that SaMD’s innovation process can be described by the innovativeness of intended use and level of integration with complementary devices. Disruptive innovations prosper in SaMD only when new entrants can build their own data sets or access data stored in the health care system. We offer recommendations that governments and academic institutions should facilitate data accessibility as a public good to accelerate innovation in SaMD.

## Appendix

Multimedia Appendix 1 Additional graphs and tables.

Multimedia Appendix 2 Number of US Food and Drug Administration (FDA)-approved software as a medical device (SaMD) from 2012 to 2021. Dark bars indicate SaMD without artificial intelligence or machine learning algorithms, light bars indicate SaMD based on AI or ML.

## Abbreviations

AI: artificial intelligence
FDA: US Food and Drug Administration
GE: General Electric Company
IMDRF: International Medical Device Regulators Forum
LINAC: linear accelerator
mHealth: mobile health
ML: machine learning
MRI: magnetic resonance imaging
SaMD: software as a medical device
SIPOC: suppliers, inputs, process, outputs, and customers

## References

[1] IMDRF SaMD Working Group (2013). Software as a medical device (SaMD): key definitions. International Medical Device Regulators Forum.

[2] (2022). Digital health terms. US Food and Drug Administration.

[3] (2022). Ageing and health. World Health Organization.

[4] (2018). 4th study group on promoting private investment for utilization of health and medical information, secretariat explanatory material. Healthcare Industry Division, Ministry of Economy, Trade and Industry.

[5] Meister S, Deiters W, Becker S (2016). Digital health and digital biomarkers-enabling value chains on health data. Curr Dir Biomed Eng.

[6] (2020). What is digital health. US Food and Drug Administration.

[7] Dorn SD (2015). Digital health: hope, hype, and Amara's law. Gastroenterology.

[8] (2022). mHealth: new horizons for health through mobile technologies: second global survey on eHealth. World Health Organization.

[9] Sven M, Wolfgang D, Klausing H, Wichert R (2015). Information logistics solutions to cope with big data challenges in AAL and telemedicine. Ambient Assisted Living: 7. AAL-Kongress 2014 Berlin, Germany, January 21-22, 2014.

[10] Medical Device Research and Development Division, Medical Devices and Healthcare Division, Japan Agency for Medical Research and Development (2022). Survey on trend of software as medical device. MEDIC.

[11] Stern AD, Gordon WJ, Landman AB, Kramer DB (2019). Cybersecurity features of digital medical devices: an analysis of FDA product summaries. BMJ Open.

[12] Martinez JB (2018). Medical device security in the IoT age.

[13] Deora S, Dandekar A Quality, timeliness and reliability in software medical devices-experience.

[14] Caffery FM, Burton J, Richardson I (2010). Risk management capability model for the development of medical device software. Softw Qual J.

[15] Wu E, Wu K, Daneshjou R, Ouyang D, Ho DE, Zou J (2021). How medical AI devices are evaluated: limitations and recommendations from an analysis of FDA approvals. Nat Med.

[16] Gerke S, Babic B, Evgeniou T, Cohen IG (2020). The need for a system view to regulate artificial intelligence/machine learning-based software as medical device. NPJ Digit Med.

[17] Aisu N, Miyake M, Takeshita K, Akiyama M, Kawasaki R, Kashiwagi K, Sakamoto T, Oshika T, Tsujikawa A (2022). Regulatory-approved deep learning/machine learning-based medical devices in Japan as of 2020: a systematic review. PLOS Digit Health.

[18] Muehlematter UJ, Daniore P, Vokinger KN (2021). Approval of artificial intelligence and machine learning-based medical devices in the USA and Europe (2015-20): a comparative analysis. Lancet Digit Health.

[19] Benjamens S, Dhunnoo P, Meskó B (2020). The state of artificial intelligence-based FDA-approved medical devices and algorithms: an online database. NPJ Digit Med.

[20] (2022). 510(k) premarket notification. US Food and Drug Administration.

[21] Premarket Approval (PMA). US Food and Drug Administration.

[22] Device classification under section 513(f)(2)(De Novo). US Food and Drug Administration.

[23] Crunchbase.

[24] Quick search: co profile news. Bloomberg.

[25] The information you need to win. PitchBook.

[26] Calinski T, Harabasz J (1974). A dendrite method for cluster analysis. Comm Statist.

[27] Hartigan JA (1975). Clustering Algorithms.

[28] Krzanowski WJ, Lai YT (1988). A criterion for determining the number of groups in a data set using sum-of-squares clustering. Biometrics.

[29] Deming WE (2000). Out of the Crisis.

[30] Deming WE (1981). Improvement of quality and productivity through action by management. Natl Prod Rev.

[31] Scholtes Pr (1998). The Leader's Handbook: Making Things Happen, Getting Things Done.

[32] Senge PM (2006). The Fifth Discipline: The Art and Practice of the Learning Organization.

[33] Onodera R, Sengoku S (2018). Innovation process of mHealth: an overview of FDA-approved mobile medical applications. Int J Med Inform.

[34] Hechavarria DM, Ingram A (2014). A review of the entrepreneurial ecosystem and the entrepreneurial society in the United States: an exploration with the global entrepreneurship monitor dataset. J Bus Entrep.

[35] (2022). Q3 2022 digital health funding: the market isn’t the same as it was. RockHealth.

[36] Bower JL, Christensen CM (1995). Disruptive technologies: catching the wave: Bower, J. L. and Christensen, C. M. harvard business review 73 (1), 43–53 (Jan–Feb 1995). Long Range Plann.

[37] Nishitani H (2007). Clinical significance and problems of three dimensional CT data: clinical practice overwhelmed by huge 3DCT data. Med Imaging Technol.

[38] Hiroshi F, Takayuki I, Shigehiko K, Takeshi H, Yoshito M, Akiko K, Hideaki H (2012). Handbook of Practical Image Analysis in Medicine.

[39] Christensen CM, Grossman JH, Hwang J (2009). The Innovator's Prescription: A Disruptive Solution for Health Care.

[40] (2021). Siemens healthineers completes acquisition of varian, strengthening its position as a holistic partner in healthcare. Varian.

[41] (2021). In an up-to $200M acquisition by Nanox, Zebra medical vision brings its AI to reimagine radiology globally. Business Wire.

[42] (2022). Expectation vs. reality: cost and time to bring SaMD to market. BrightInsight.

[43] Elenko E, Underwood L, Zohar D (2015). Defining digital medicine. Nat Biotechnol.

[44] Steinberg D, Horwitz G, Zohar D (2015). Building a business model in digital medicine. Nat Biotechnol.

[45] Wiener JB (2004). The regulation of technology, and the technology of regulation. Technol Soc.

[46] Porter ME (1980). Competitive Strategy: Techniques for Analyzing Industries and Competitors.

[47] Ulrich K (1995). The role of product architecture in the manufacturing firm. Res Policy.

[48] Sato O, Fujimura S (2016). An influence of potentiality and constraint of the physical principle involved in product architecture on competitive strategy: a case study on the competition between LCD and PDP in the flat-Panel TV market. Organ Sci.

