# Supplementary material for: Innovation Process and Industrial System of US Food and Drug Administration–Approved Software as a Medical Device: Review and Content Analysis
Source: J Med Internet Res. 2023 Nov 24;25:e47505. doi: 10.2196/47505 (PMC10709785; doi:10.2196/47505)
Supplement: Multimedia Appendix 1 [file jmir_v25i1e47505_app1.docx]

**Innovation Process and Industrial System of Software as Medical Device: A review of FDA-approved SaMD**

Jiakan Yu^1^, MA; Jiajie Zhang^2^, MA; Shintaro Sengoku^1,3^ , PhD

^1^Department of Technology and Innovation Management, School of Environment and Society, Tokyo Institute of Technology, Tokyo, Japan

^2^Graduate School of Interdisciplinary Information Studies, The University of Tokyo, Tokyo, Japan

^3^Department of Innovation Science, School of Environment and Society, Tokyo Institute of Technology, Tokyo, Japan

**Corresponding Author:**

Shintaro Sengoku, PhD

Department of Innovation Science

School of Environment and Society

Tokyo Institute of Technology

Tokyo Campus Innovation Center 9th Floor Room 908N (CIC908N)

3-3-6 Shibaura, Minato-ku Tokyo,

108-0023 Japan

Phone: 81 03 3454 8907

Email: sengoku.s.aa@m.titech.ac.jp

## Multimedia Appendix

##
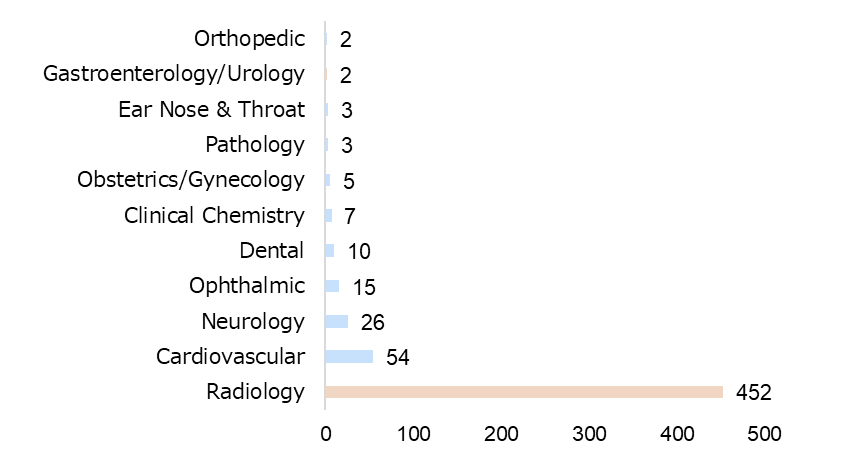


Figure S1. Number of software as a medical device (SaMD) by review panel


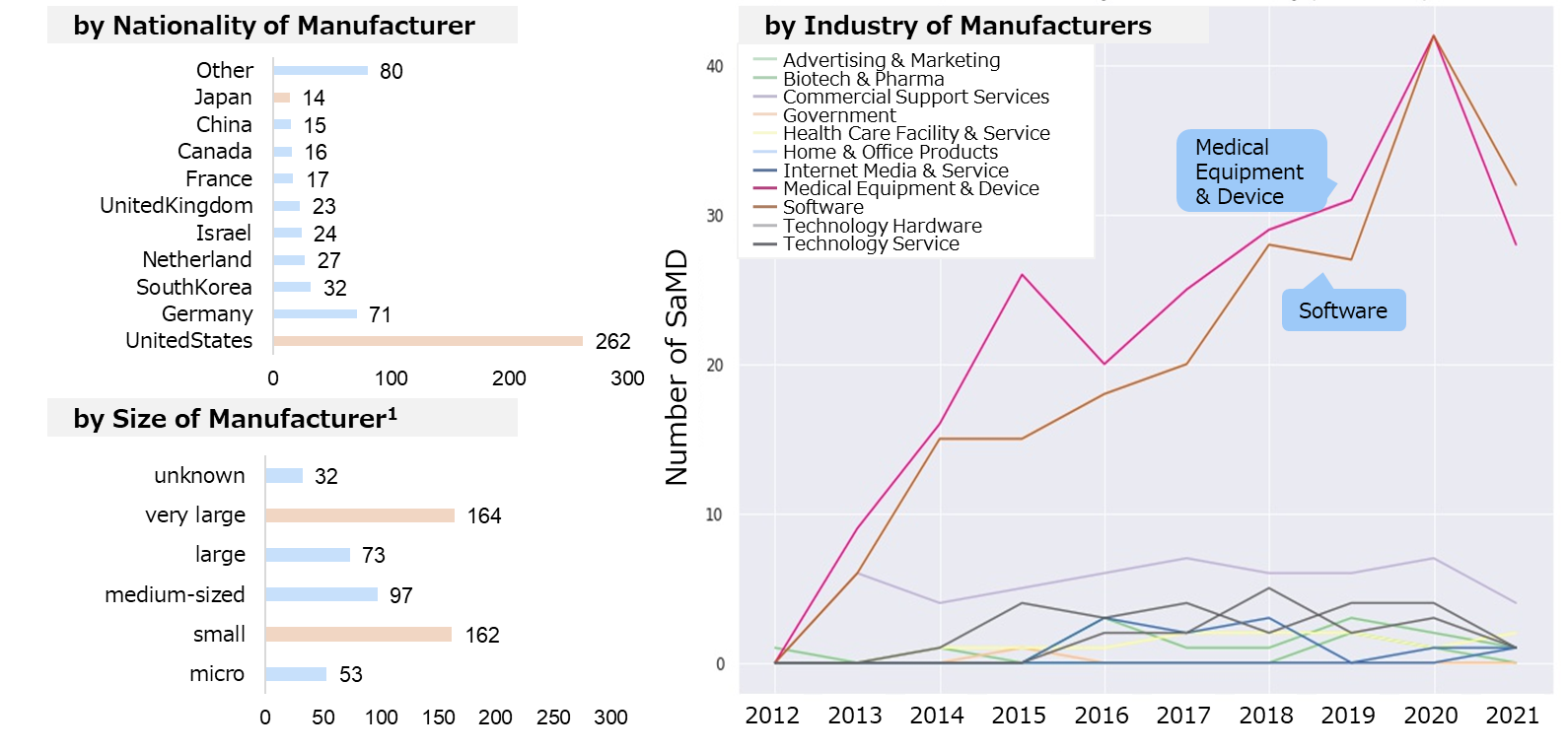


Figure S2. Number of software as a medical device (SaMD) by nationality of manufacturer


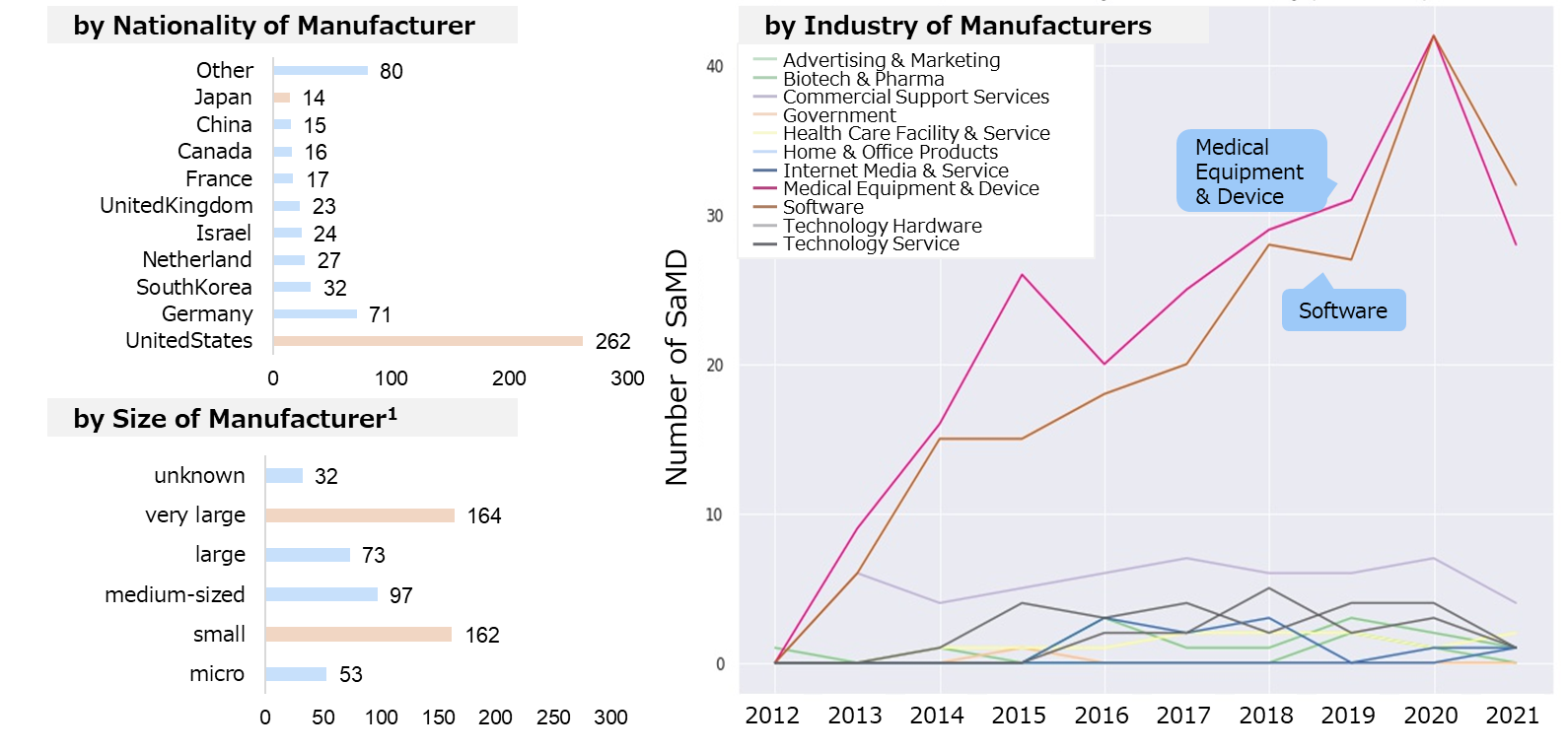


Figure S3. Number of software as a medical device (SaMD) by company size of manufacturer

Note: Enterprise is categorized by number of employees. Micro enterprise has 1-10 employees. Small enterprise has 11-50 employees. Medium-sized enterprise has 51-250 employees. Large enterprise has 251-1000 employees. Very large Enterprise has over 1001 employees. Unknow are enterprise whose data is not available.


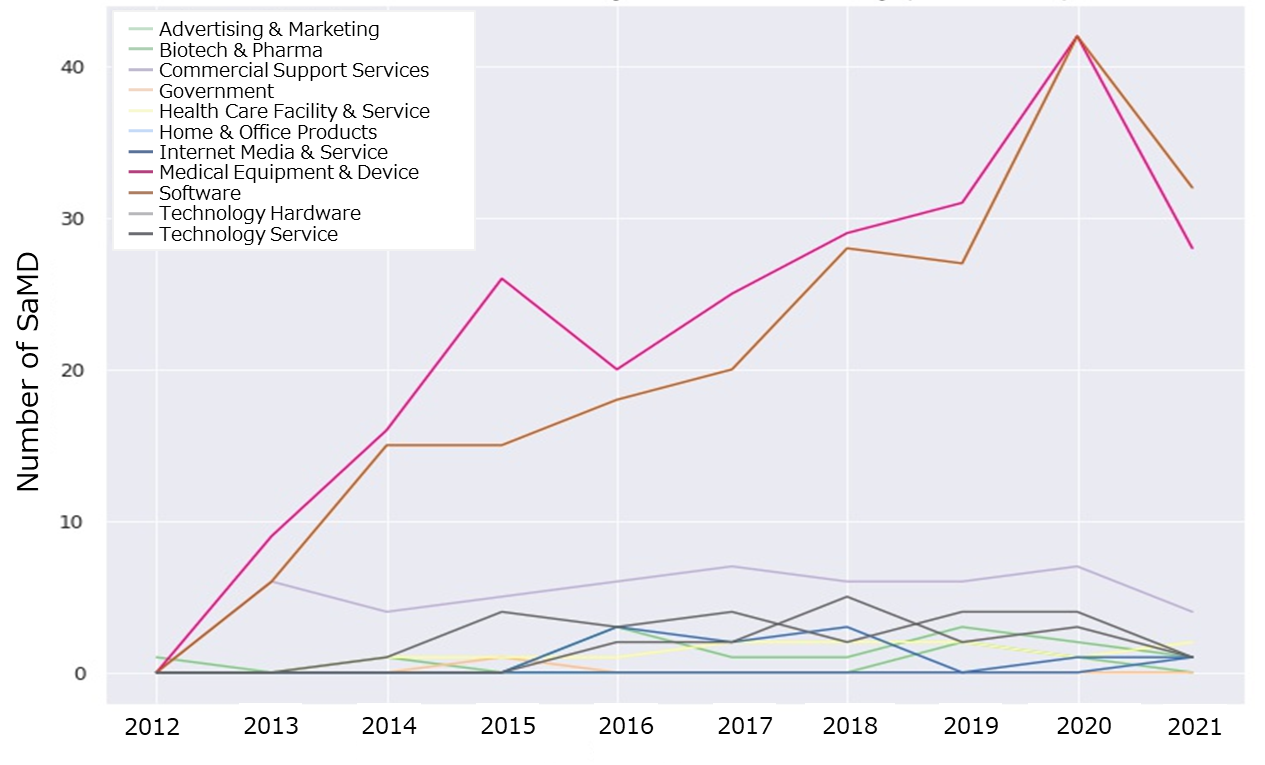


Figure S4. Number of software as a medical device (SaMD) by industry of manufacturer

Table S1. List of M&A deal in SaMD

| # | **Manufacturer Name** | **Deal happened Year** | **Acquirer** |
| --- | --- | --- | --- |
| 1 | ZOLL Medical Corporation | 2012 | Asahi Kasei |
| 2 | Spectrum-Dynamics | 2013 | Biosensors |
| 3 | Covidien | 2014 | Medtronic |
| 4 | Nobel Biocare | 2014 | Danaher |
| 5 | BioMet | 2014 | Zimmer Biomet |
| 6 | Carefusion | 2014 | Becton, Dickinson and Company |
| 7 | Virtual Radiologic Corporation | 2015 | MEDNAX |
| 8 | MeVis Medical Solutions AG | 2015 | Varian |
| 9 | Nines | 2015 | Sentia |
| 10 | Welch Allyn | 2015 | Hill-Rom |
| 11 | 20/20 Imaging | 2015 | Konica Minolta |
| 12 | Optos | 2015 | Nikon |
| 13 | Olea Medical | 2015 | Toshiba Medical |
| 14 | Haag-Streit USA | 2016 | Luneau Tech |
| 15 | Toshiba Medical Systems | 2016 | Cannon |
| 16 | PeraHealth | 2016 | Mainsail Partners |
| 17 | NeuWave Medical | 2016 | ETHICON,Inc. |
| 18 | TomTec Imaging Systems | 2017 | Philips |
| 19 | Imaging Biometrics | 2018 | Flying Brands |
| 20 | Mobius Medical Systems | 2018 | Varian |
| 21 | VidiStar | 2018 | Hitachi |
| 22 | ProKnow Systems | 2019 | Elekta |
| 23 | Client Outlook | 2020 | Mach7 Technologies |
| 24 | Therenva | 2020 | Ziehm Imaging |
| 25 | DeepHealth | 2020 | RadNet |
| 26 | EndoVantage | 2020 | RapidAI |
| 27 | Agfa HealthCare | 2020 | Dedalus Group |
| 28 | Medicrea | 2020 | Medtronic |
| 29 | exocad GmbH | 2020 | Align Technology |
| 30 | Apteryx Imaging | 2020 | Planet DDS |
| 31 | Varian | 2021 | Siemens |
| 32 | Zebra Medical Vision | 2021 | Nanox Imaging |
| 33 | Heart Imaging Technologies | 2021 | Intelerad |
| 34 | Terarecon | 2021 | ConcerAI |
| 35 | Mint Medical | 2021 | BrainLAB |
| 36 | Fitbit | 2021 | Google |
| 37 | Carestream | 2021 | Philips |
| 38 | Medusa Medical Technologies | 2021 | Emergency Reporting |
| 39 | Cardiologs | 2021 | Philips |
| 40 | Natus Medical | 2022 | ArchiMed |
| 41 | Monarch Medical Technologies | 2022 | Semler |
| 42 | Quantib | 2022 | RadNet |
| 43 | Change Healthcare | 2022 | UHG |

Table S2. Indicator to evaluate interoperability of clustering

| Number of Cluster | CH | H | KL |
| --- | --- | --- | --- |
| 2 | 331.80 | 191.08 | 2.58 |
| 3 | 369.80 | 101.71 | 2.46 |
| 4 | 366.05 | 93.34 | 1.34 |
| 5 | 385.59 | 42.21 | 2.70 |
| 6 | 361.05 | 47.27 | 0.95 |
| 7 | 357.26 | 48.35 | 1.08 |
| 8 | 363.83 | 37.95 | 1.43 |
| 9 | 364.37 | 36.08 | 1.15 |
| 10 | 367.90 | 31.98 | 1.23 |

Note: CH means Calinski-Harabasz index. H means Hartigan index. KL means Krzanowski-Lai index.

**
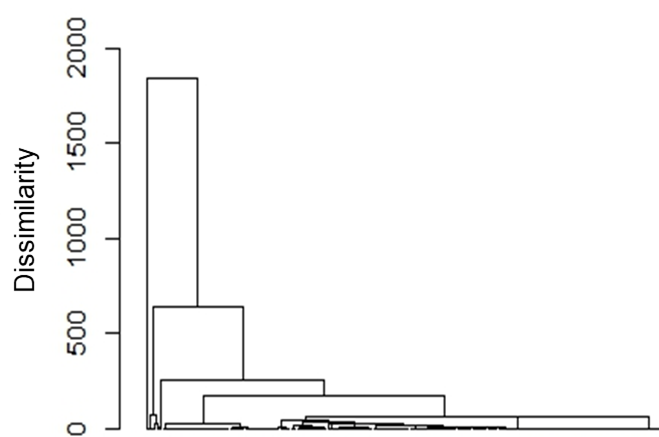
Figure S5. Ward-linkage cluster dendrogram**

Table S3. Eigenvalue by dimension

| Dimension | Eigenvalue | Percentage of Variance | Cumulative Percentage of Variance |
| --- | --- | --- | --- |
| dim1 | 0.402 | 61.731 % | 61.731 % |
| dim2 | 0.232 | 35.600 % | 97.331 % |
| dim3 | 0.014 | 2.093 % | 99.425 % |
| dim4 | 0.004 | 0.575 % | 100.000 % |

Table S4. Results of the correspondence analysis

Columns

| Medical Specialty | Iner ×1000 | Dim1 | | | Dim2 | | |
| --- | --- | --- | --- | --- | --- | --- | --- |
|  |  | coord | ctr | cos2 | coord | ctr | cos2 |
| clinical data managing | 21.69 | 0.35 | 0.88 | 0.16 | 0.79 | 7.80 | 0.83 |
| diagnostic evaluation | 37.36 | 0.24 | 1.04 | 0.11 | 0.63 | 12.53 | 0.78 |
| digital therapeutics | 6.45 | 0.40 | 0.28 | 0.18 | 0.87 | 2.26 | 0.81 |
| image capture | 8.06 | 0.40 | 0.35 | 0.18 | 0.87 | 2.83 | 0.81 |
| image processing, analysis | 113.98 | -0.36 | 20.74 | 0.73 | -0.22 | 13.15 | 0.27 |
| image viewing and management | 34.06 | 0.35 | 1.34 | 0.16 | 0.80 | 11.99 | 0.82 |
| lesion identification | 13.35 | 0.02 | 0.00 | 0.00 | 0.30 | 0.80 | 0.14 |
| rehabilitation support | 1.61 | 0.40 | 0.07 | 0.18 | 0.87 | 0.57 | 0.81 |
| remote monitoring | 23.25 | 0.35 | 0.94 | 0.16 | 0.79 | 8.36 | 0.83 |
| review, monitoring and analysis | 4.84 | 0.40 | 0.21 | 0.18 | 0.87 | 1.70 | 0.81 |
| therapy decision support | 14.51 | 0.40 | 0.63 | 0.18 | 0.87 | 5.09 | 0.81 |
| therapy treatment planning | 336.89 | 1.95 | 71.97 | 0.86 | -0.79 | 20.46 | 0.14 |
| visualization, measurement, simulation | 35.47 | 0.40 | 1.54 | 0.18 | 0.87 | 12.45 | 0.81 |

Note: Iner means inertia for the columns. Coord means the coordinate for columns. Ctr means contribution of the columns. Cos2 means squared correlation for the columns.

Row

| Cluster | Iner ×1000 | Dim1 | | | Dim2 | | |
| --- | --- | --- | --- | --- | --- | --- | --- |
|  |  | coord | ctr | cos2 | coord | ctr | cos2 |
| 1 | 125.45 | 0.26 | 8.47 | 0.27 | 0.42 | 39.39 | 0.73 |
| 2 | 113.84 | -0.55 | 17.70 | 0.63 | -0.41 | 17.68 | 0.36 |
| 3 | 35.95 | -0.37 | 4.58 | 0.51 | -0.29 | 4.94 | 0.32 |
| 4 | 40.18 | -0.45 | 4.19 | 0.42 | -0.41 | 5.92 | 0.34 |
| 5 | 336.10 | 3.08 | 65.07 | 0.78 | -1.64 | 32.08 | 0.22 |

Note: Iner means inertia for the row. Coord means the coordinate for rows. Ctr means contribution of the rows. Cos2 means squared correlation for the rows.

Table S5. Distribution of software as a medical device (SaMD) by medical specialty and device classification

| **Medical Specialty** | **Device Classification** | **Pattern1** | **Pattern2** | **Pattern3** | **Total** |
| --- | --- | --- | --- | --- | --- |
| Radiology | Accelerator, Linear, Medical |  | 17 |  | 17 |
|  | Analyzer, Medical Image |  | 1 |  | 1 |
|  | Automated Radiological Image Processing Software |  | 15 |  | 15 |
|  | Colon Computed Tomography System, Computer Aided Detection |  | 1 |  | 1 |
|  | Computer-Assisted Diagnostic Software For Lesions Suspicious For Cancer |  | 6 |  | 6 |
|  | Image Management Software For Planning Of Otologic And Neurotologic Procedures | 1 |  |  | 1 |
|  | Interventional Fluoroscopic X-Ray System | 2 |  |  | 2 |
|  | Liver Iron Concentration Imaging Companion Diagnostic For Deferasirox |  | 2 |  | 2 |
|  | Lung Computed Tomography System, Computer-Aided Detection |  | 3 |  | 3 |
|  | Radiological Computer Assisted Detection/Diagnosis Software For Fracture |  | 2 |  | 2 |
|  | Radiological Computer Assisted Detection/Diagnosis Software For Lesions Suspicious For Cancer |  | 12 |  | 12 |
|  | Radiological Computer-Assisted Prioritization Software For Lesions |  | 12 |  | 12 |
|  | Radiological Computer-Assisted Triage And Notification Software |  | 22 |  | 22 |
|  | Radiological Image Processing Software For Radiation Therapy |  | 10 |  | 10 |
|  | Software For Visualization Of Vascular Anatomy And Intravascular Devices |  | 1 |  | 1 |
|  | Solid State X-Ray Imager (Flat Panel/Digital Imager) | 5 |  |  | 5 |
|  | System, Image Management, Ophthalmic | 14 |  |  | 14 |
|  | System, Image Processing, Radiological |  | 266 |  | 266 |
|  | System, Imaging, Pulsed Doppler, Ultrasonic |  | 1 |  | 1 |
|  | System, Imaging, Pulsed Echo, Ultrasonic | 1 |  |  | 1 |
|  | System, Nuclear Magnetic Resonance Imaging |  | 8 |  | 8 |
|  | System, Planning, Radiation Therapy Treatment | 24 |  |  | 24 |
|  | System, Simulation, Radiation Therapy | 1 |  |  | 1 |
|  | System, X-Ray, Stationary | 1 |  |  | 1 |
|  | System, X-Ray, Tomography, Computed |  | 38 |  | 38 |
|  | X-Ray Angiographic Imaging Based Coronary Vascular Simulation Software Device | 2 |  |  | 2 |
| Physical Medicine | Interactive Rehabilitation Exercise Devices |  |  | 1 | 1 |
| Pathology | Digital Pathology Image Viewing And Management Software | 2 |  |  | 2 |
|  | Software Algorithm Device To Assist Users In Digital Pathology |  | 1 |  | 1 |
| Orthopedic | Prosthesis, Shoulder, Semi-Constrained, Metal/Polymer Cemented |  | 2 |  | 2 |
| Ophthalmic | Digital Therapy Device For Amblyopia |  |  | 1 | 1 |
| Obstetrics/Gynecology | Device, Fertility Diagnostic, Contraceptive, Software Application |  |  | 1 | 1 |
|  | System, Monitoring, Perinatal | 4 |  |  | 4 |
| Neurology | Amplitude-Integrated Electroencephalograph | 3 |  |  | 3 |
|  | Automatic Event Detection Software For Full-Montage Electroencephalograph |  | 3 |  | 3 |
|  | Automatic Event Detection Software For Polysomnograph With Electroencephalograph |  |  | 10 | 10 |
|  | Computerized Cognitive Assessment Aid For Concussion |  | 1 |  | 1 |
|  | Neurological Stereotaxic Instrument |  | 2 |  | 2 |
|  | Non-Normalizing Quantitative Electroencephalograph Software | 2 |  |  | 2 |
|  | Normalizing Quantitative Electroencephalograph Software |  | 3 |  | 3 |
|  | Source Localization Software For Electroencephalograph Or Magnetoencephalograph |  | 2 |  | 2 |
| Gastroenterology/Urology | Computerized Behavioral Therapy Device For Treating Symptoms |  |  | 1 | 1 |
|  | Gastrointesinal Lesion Software Detection System |  | 1 |  | 1 |
| Ear Nose & Throat | Apparatus, Vestibular Analysis |  |  | 1 | 1 |
|  | Masker, Tinnitus |  |  | 1 | 1 |
| Dental | Dental Abutment Design Software For Dental Laboratory |  | 3 |  | 3 |
|  | Orthodontic Software |  | 7 |  | 7 |
| Clinical Chemistry | Insulin Pump Therapy Adjustment Calculator For Healthcare Professionals | 2 |  |  | 2 |
| Cardiovascular | Adjunctive Hemodynamic Indicator With Decision Point |  |  | 1 | 1 |
|  | Angiographic Coronary Vascular Physiologic Simulation Software |  | 2 |  | 2 |
|  | Computer, Diagnostic, Programmable |  | 9 |  | 9 |
|  | Coronary Vascular Physiologic Simulation Software |  | 6 |  | 6 |
|  | Detector And Alarm, Arrhythmia |  |  | 1 | 1 |
|  | Display, Cathode-Ray Tube, Medical | 2 |  |  | 2 |
|  | Electrocardiograph |  |  | 6 | 6 |
|  | Electrocardiograph Software For Over-The-Counter Use |  |  | 1 | 1 |
|  | Interventional Cardiovascular Implant Simulation Software Device |  | 1 |  | 1 |
|  | Medium-Term Adjunctive Predictive Cardiovascular Indicator |  | 1 |  | 1 |
|  | Monitor, Physiological, Patient (Without Arrhythmia Detection Or Alarms) | 10 |  |  | 10 |
|  | Monitor, Physiological, Patient(With Arrhythmia Detection Or Alarms) |  |  | 3 | 3 |
|  | Multivariate Vital Signs Index |  |  | 3 | 3 |
|  | Photoplethysmograph Analysis Software For Over-The-Counter Use |  |  | 2 | 2 |
|  | System, Network And Communication, Physiological Monitors |  |  | 4 | 4 |
|  | Transmitters And Receivers, Physiological Signal, Radiofrequency |  |  | 1 | 1 |
| Anesthesiology | Calculator, Drug Dose | 5 |  |  | 5 |
|  | Ventilatory Effort Recorder | 1 |  |  | 1 |
| **Total** | | 82 | 461 | 38 | 581 |

Table S6. Contingency table of number of software as a medical device (SaMD) by manufacturers and medical specialty (Item 1-7)

| **Manufacturer Name** | **clinical data managing** | **diagnostic evaluation** | **digital therapeutics** | **image capture** | **image processing, analysis** | **image viewing and management** | **lesion identification** |
| --- | --- | --- | --- | --- | --- | --- | --- |
| 20/20 Imaging | 0 | 0 | 0 | 1 | 0 | 0 | 0 |
| 3D Industrial Imaging | 0 | 0 | 0 | 0 | 2 | 0 | 0 |
| 3D Systems | 0 | 0 | 0 | 0 | 2 | 0 | 0 |
| 3Shape | 0 | 0 | 0 | 0 | 2 | 0 | 0 |
| 4DX | 0 | 0 | 0 | 0 | 1 | 0 | 0 |
| Ablacon | 0 | 1 | 0 | 0 | 0 | 0 | 0 |
| Acuitas Medical | 0 | 0 | 0 | 0 | 1 | 0 | 0 |
| ADAS3D Medical | 0 | 0 | 0 | 0 | 2 | 0 | 0 |
| ADM Diagnostics | 0 | 0 | 0 | 0 | 1 | 0 | 0 |
| Advanced Brain Monitoring | 0 | 1 | 0 | 0 | 0 | 0 | 0 |
| Advantis Medical Imaging | 0 | 0 | 0 | 0 | 1 | 0 | 0 |
| Agfa HealthCare | 0 | 0 | 0 | 0 | 2 | 0 | 0 |
| AI Metrics | 0 | 0 | 0 | 0 | 1 | 0 | 0 |
| AIbolit Technologies | 0 | 0 | 0 | 0 | 1 | 0 | 0 |
| Aidoc | 0 | 0 | 0 | 0 | 8 | 0 | 0 |
| AIRS Medical | 0 | 0 | 0 | 0 | 1 | 0 | 0 |
| Airstrip Technologies | 1 | 0 | 0 | 0 | 0 | 0 | 0 |
| AIT Austrian Institute Of Technology | 0 | 0 | 0 | 0 | 0 | 0 | 0 |
| Al Pacheco | 0 | 0 | 0 | 0 | 0 | 0 | 0 |
| AlertWatch | 1 | 0 | 0 | 0 | 0 | 0 | 0 |
| Algomedica | 0 | 0 | 0 | 0 | 1 | 0 | 0 |
| AliveCor | 0 | 2 | 0 | 0 | 0 | 0 | 0 |
| Alphatec Spine | 0 | 0 | 0 | 0 | 1 | 0 | 0 |
| AmCad BioMed | 0 | 0 | 0 | 0 | 2 | 0 | 0 |
| Anatomage Inc. | 0 | 0 | 0 | 0 | 2 | 0 | 0 |
| Apollo Medical Imaging Technology | 0 | 0 | 0 | 0 | 1 | 0 | 0 |
| Apple | 0 | 0 | 0 | 0 | 0 | 0 | 0 |
| Apteryx Imaging | 0 | 0 | 0 | 0 | 1 | 0 | 0 |
| ArchForm | 0 | 0 | 0 | 0 | 0 | 0 | 0 |
| ArteryFlow | 0 | 0 | 0 | 0 | 0 | 1 | 0 |
| Arterys | 0 | 0 | 0 | 0 | 1 | 0 | 0 |
| ArthroMeda | 0 | 0 | 0 | 0 | 1 | 0 | 0 |
| ASCEND HiT | 0 | 0 | 0 | 0 | 1 | 0 | 0 |
| Austrian Institute of Technology | 0 | 0 | 0 | 0 | 0 | 0 | 0 |
| Avicenna.ai | 0 | 0 | 0 | 0 | 2 | 0 | 0 |
| Behold.ai | 0 | 0 | 0 | 0 | 1 | 0 | 0 |
| Best NOMOS | 0 | 0 | 0 | 0 | 0 | 0 | 0 |
| Biobot Surgical Pte Ltd | 0 | 0 | 0 | 0 | 1 | 0 | 0 |
| Biofourmis | 0 | 0 | 0 | 0 | 0 | 0 | 0 |
| Biolight | 1 | 1 | 0 | 0 | 0 | 0 | 0 |
| BioMet | 0 | 0 | 0 | 0 | 1 | 0 | 0 |
| BoxView | 0 | 0 | 0 | 0 | 0 | 0 | 0 |
| BrainLAB | 0 | 0 | 0 | 0 | 2 | 0 | 0 |
| BrainMaster Technologies | 0 | 1 | 0 | 0 | 0 | 0 | 0 |
| BrainNow | 0 | 0 | 0 | 0 | 1 | 0 | 0 |
| Brainomix | 0 | 0 | 0 | 0 | 1 | 0 | 0 |
| Broncus Technologies | 0 | 0 | 0 | 0 | 1 | 0 | 0 |
| B-Secur | 0 | 0 | 0 | 0 | 0 | 0 | 0 |
| Cadwell Laboratories | 0 | 1 | 0 | 0 | 0 | 1 | 0 |
| Calgary Scientific | 0 | 0 | 0 | 0 | 1 | 0 | 0 |
| Canon Medical Systems | 0 | 0 | 0 | 0 | 4 | 1 | 0 |
| Caption Health | 0 | 0 | 0 | 0 | 1 | 0 | 0 |
| Cardiologs | 0 | 0 | 0 | 0 | 0 | 0 | 0 |
| Carefusion | 0 | 1 | 0 | 0 | 0 | 0 | 0 |
| Carestream | 0 | 0 | 0 | 1 | 1 | 0 | 0 |
| Caristo Diagnostics | 0 | 0 | 0 | 0 | 1 | 0 | 0 |
| Carl Zeiss Meditec | 0 | 0 | 0 | 0 | 0 | 3 | 0 |
| CAScination | 0 | 0 | 0 | 0 | 0 | 1 | 0 |
| CathWorks | 0 | 0 | 0 | 0 | 0 | 0 | 0 |
| CDB Corp | 0 | 0 | 0 | 0 | 0 | 0 | 0 |
| Ceevra, Inc. | 0 | 0 | 0 | 0 | 2 | 0 | 0 |
| Change Healthcare | 0 | 0 | 0 | 0 | 1 | 0 | 0 |
| Circle Cardiovascular Imaging | 0 | 0 | 0 | 0 | 3 | 0 | 0 |
| ClariPi | 0 | 0 | 0 | 0 | 2 | 0 | 0 |
| Claritas HealthTech | 0 | 0 | 0 | 0 | 1 | 0 | 0 |
| ClearView Diagnostics | 0 | 0 | 0 | 0 | 1 | 0 | 0 |
| Cleerly | 0 | 0 | 0 | 0 | 2 | 0 | 0 |
| CLEW Medical | 0 | 0 | 0 | 0 | 0 | 0 | 0 |
| Client Outlook | 0 | 0 | 0 | 0 | 1 | 0 | 0 |
| CONSOLIDATED RESEARCH | 0 | 1 | 0 | 0 | 0 | 0 | 0 |
| Cordiana Medical Informatics | 0 | 1 | 0 | 0 | 0 | 0 | 0 |
| CORELINESOFT | 0 | 0 | 0 | 0 | 2 | 0 | 0 |
| CorTechs Labs | 0 | 0 | 0 | 0 | 1 | 0 | 0 |
| CorticoMetrics | 0 | 0 | 0 | 0 | 1 | 0 | 0 |
| Covidien | 1 | 0 | 0 | 0 | 0 | 0 | 0 |
| Cuptimize | 0 | 0 | 0 | 0 | 1 | 0 | 0 |
| CureMetrix | 0 | 0 | 0 | 0 | 1 | 0 | 0 |
| Curve Beam | 0 | 0 | 0 | 0 | 1 | 0 | 0 |
| Cycle Clarity | 0 | 0 | 0 | 0 | 1 | 0 | 0 |
| Cydar Medical | 0 | 0 | 0 | 0 | 0 | 2 | 0 |
| de Gotzen | 0 | 0 | 0 | 0 | 1 | 0 | 0 |
| Decisio Health | 1 | 0 | 0 | 0 | 0 | 0 | 0 |
| Deep01 Technology | 0 | 0 | 0 | 0 | 1 | 0 | 0 |
| DeepHealth | 0 | 0 | 0 | 0 | 1 | 0 | 0 |
| DeepLook | 0 | 0 | 0 | 0 | 1 | 0 | 0 |
| Deepvoxel | 0 | 0 | 0 | 0 | 1 | 0 | 0 |
| Densitas | 0 | 0 | 0 | 0 | 2 | 0 | 0 |
| Dentsply Sirona | 0 | 0 | 0 | 0 | 1 | 0 | 0 |
| Dexin Medical Imaging Technology | 0 | 0 | 0 | 0 | 1 | 0 | 0 |
| Digital Core Technology | 0 | 0 | 0 | 0 | 1 | 0 | 0 |
| DOSIsoft | 0 | 0 | 0 | 0 | 1 | 0 | 0 |
| DreaMed Diabetes | 0 | 0 | 0 | 0 | 0 | 0 | 0 |
| DRGEM | 0 | 0 | 0 | 0 | 1 | 0 | 0 |
| Dürr Dental | 0 | 0 | 0 | 0 | 4 | 0 | 0 |
| EB Neuro | 0 | 0 | 0 | 0 | 0 | 1 | 0 |
| EBM Technologies | 0 | 0 | 0 | 0 | 2 | 0 | 0 |
| EchoPixel | 0 | 0 | 0 | 0 | 1 | 0 | 0 |
| Eckert & Ziegler Bebig SA | 0 | 0 | 0 | 0 | 0 | 0 | 0 |
| Edan Instruments | 2 | 0 | 0 | 0 | 0 | 0 | 0 |
| EDDA Technology | 0 | 0 | 0 | 0 | 1 | 0 | 0 |
| eko.ai | 0 | 0 | 0 | 0 | 1 | 0 | 0 |
| Elekta | 0 | 0 | 0 | 0 | 0 | 0 | 0 |
| Eli Lilly | 0 | 0 | 0 | 0 | 0 | 0 | 0 |
| ElMindA | 0 | 2 | 0 | 0 | 0 | 0 | 0 |
| Elucid | 0 | 0 | 0 | 0 | 2 | 0 | 0 |
| EndoVantage | 0 | 0 | 0 | 0 | 0 | 0 | 0 |
| EnsoData | 0 | 2 | 0 | 0 | 0 | 0 | 0 |
| Epilog | 0 | 1 | 0 | 0 | 0 | 0 | 0 |
| Epiphany Healthcare | 0 | 1 | 0 | 0 | 0 | 0 | 0 |
| Etiometry | 5 | 0 | 0 | 0 | 0 | 0 | 0 |
| Ever Fortune.AI | 0 | 0 | 0 | 0 | 1 | 0 | 0 |
| eVolve PACS | 0 | 0 | 0 | 0 | 1 | 0 | 0 |
| Ewoosoft | 0 | 0 | 0 | 0 | 15 | 0 | 0 |
| EXINI Diagnostics | 0 | 0 | 0 | 0 | 2 | 0 | 0 |
| exocad GmbH | 0 | 0 | 0 | 0 | 1 | 0 | 0 |
| FEops | 0 | 0 | 0 | 0 | 0 | 0 | 0 |
| Fifth Eye | 0 | 0 | 0 | 0 | 0 | 0 | 0 |
| Firstsource | 0 | 0 | 0 | 1 | 0 | 0 | 0 |
| Fitbit | 0 | 0 | 0 | 0 | 0 | 0 | 0 |
| FLUIDDA | 0 | 0 | 0 | 0 | 1 | 0 | 0 |
| Fujifilm Medical Systems | 0 | 0 | 0 | 0 | 5 | 0 | 0 |
| Galgo Medical | 0 | 0 | 0 | 0 | 1 | 0 | 0 |
| GE Healthcare | 0 | 5 | 0 | 0 | 17 | 0 | 0 |
| Genesis Software Innovations | 0 | 0 | 0 | 0 | 1 | 0 | 0 |
| GMV | 0 | 0 | 0 | 0 | 0 | 0 | 0 |
| Haag-Streit USA | 0 | 0 | 0 | 0 | 0 | 1 | 0 |
| Halifax Biomedical | 0 | 0 | 0 | 0 | 1 | 0 | 0 |
| HDX WILL | 0 | 0 | 0 | 0 | 3 | 0 | 0 |
| HealthMyne | 0 | 0 | 0 | 0 | 2 | 0 | 0 |
| Heart Imaging Technologies | 0 | 0 | 0 | 0 | 1 | 0 | 0 |
| HeartFlow | 0 | 0 | 0 | 0 | 0 | 0 | 0 |
| Highmark Interactive | 0 | 0 | 1 | 0 | 0 | 0 | 0 |
| Hinacom | 0 | 0 | 0 | 0 | 1 | 0 | 0 |
| Hologic | 0 | 0 | 0 | 0 | 1 | 0 | 0 |
| iCAD | 0 | 0 | 0 | 0 | 3 | 0 | 0 |
| icometrix | 0 | 0 | 0 | 0 | 2 | 0 | 0 |
| ImageBiopsy Lab | 0 | 0 | 0 | 0 | 1 | 0 | 0 |
| Imagen Technologies | 0 | 0 | 0 | 0 | 3 | 0 | 0 |
| Imaging Biometrics | 0 | 0 | 0 | 0 | 1 | 0 | 0 |
| Imaging Sciences International | 0 | 0 | 0 | 0 | 2 | 0 | 0 |
| Imbio | 0 | 0 | 0 | 0 | 1 | 0 | 0 |
| IMPAC Medical System | 0 | 0 | 0 | 0 | 0 | 0 | 0 |
| Infervision | 0 | 0 | 0 | 0 | 1 | 0 | 0 |
| Infinitt | 0 | 0 | 0 | 0 | 3 | 0 | 0 |
| Intelligent Retinal Imaging Systems | 0 | 0 | 0 | 0 | 0 | 1 | 0 |
| Intellijoint Surgical | 0 | 0 | 0 | 0 | 1 | 0 | 0 |
| International Medical Solutions | 0 | 0 | 0 | 0 | 1 | 0 | 0 |
| Intoware | 0 | 0 | 0 | 0 | 1 | 0 | 0 |
| Intuitive Surgical | 0 | 0 | 0 | 0 | 1 | 0 | 0 |
| InVivo Therapeutics | 0 | 0 | 0 | 0 | 2 | 0 | 0 |
| Jintronix | 0 | 0 | 0 | 0 | 0 | 0 | 0 |
| JointVue | 0 | 0 | 0 | 0 | 1 | 0 | 0 |
| JPI Healthcare Solutions | 0 | 0 | 0 | 0 | 1 | 0 | 0 |
| KEYA MEDICAL | 0 | 0 | 0 | 0 | 1 | 0 | 0 |
| Koios Medical | 0 | 0 | 0 | 0 | 0 | 0 | 2 |
| LEAD Technologies, Inc. | 0 | 0 | 0 | 0 | 1 | 0 | 0 |
| Lexi | 0 | 0 | 0 | 0 | 2 | 0 | 0 |
| Limbus AI | 0 | 0 | 0 | 0 | 1 | 0 | 0 |
| LK Consulting | 0 | 0 | 0 | 0 | 1 | 0 | 0 |
| Luminopia | 0 | 0 | 1 | 0 | 0 | 0 | 0 |
| Lunit | 0 | 0 | 0 | 0 | 2 | 0 | 0 |
| Materialise | 0 | 0 | 0 | 0 | 1 | 0 | 0 |
| MATH RESOLUTIONS | 0 | 0 | 0 | 0 | 0 | 0 | 0 |
| MaxQ-AI | 0 | 0 | 0 | 0 | 2 | 0 | 0 |
| McKesson | 0 | 0 | 0 | 0 | 1 | 0 | 0 |
| Medic Vision | 0 | 0 | 0 | 0 | 1 | 0 | 0 |
| MedicalCommunications | 0 | 0 | 0 | 0 | 1 | 0 | 0 |
| Medicrea | 0 | 0 | 0 | 0 | 2 | 0 | 0 |
| Medis Medical Imaging Systems BV | 0 | 0 | 0 | 0 | 0 | 1 | 0 |
| MEDIVIS | 0 | 0 | 0 | 0 | 1 | 0 | 0 |
| Medmind | 0 | 0 | 0 | 0 | 1 | 0 | 0 |
| Medo.ai | 0 | 0 | 0 | 0 | 1 | 0 | 0 |
| Medsynaptic | 0 | 0 | 0 | 0 | 1 | 0 | 0 |
| Medusa Medical Technologies | 1 | 0 | 0 | 0 | 0 | 0 | 0 |
| Medviso | 0 | 0 | 0 | 0 | 1 | 0 | 0 |
| Merge | 0 | 0 | 0 | 0 | 3 | 0 | 0 |
| metaMe Health | 0 | 0 | 1 | 0 | 0 | 0 | 0 |
| MetriTrack | 0 | 0 | 0 | 0 | 0 | 1 | 0 |
| MeVis Medical Solutions AG | 0 | 0 | 0 | 0 | 0 | 0 | 1 |
| Microsoft | 0 | 0 | 0 | 0 | 1 | 0 | 0 |
| Mint Medical | 0 | 0 | 0 | 0 | 1 | 0 | 0 |
| Mirada Medical | 0 | 1 | 0 | 0 | 3 | 0 | 0 |
| Mobius Medical Systems | 0 | 0 | 0 | 0 | 0 | 0 | 0 |
| Monarch Medical Technologies | 0 | 0 | 0 | 0 | 0 | 0 | 0 |
| Motilent | 0 | 0 | 0 | 0 | 1 | 0 | 0 |
| MRIguidance | 0 | 0 | 0 | 0 | 1 | 0 | 0 |
| MultiModal Health | 0 | 0 | 0 | 0 | 1 | 0 | 0 |
| Myocardial Solutions | 0 | 1 | 0 | 0 | 0 | 0 | 0 |
| Natural Cycles | 0 | 0 | 0 | 0 | 0 | 0 | 0 |
| Natus Medical | 0 | 5 | 0 | 0 | 0 | 0 | 0 |
| NEOLOGICA | 0 | 0 | 0 | 0 | 1 | 0 | 0 |
| NeuroChaos Solutions | 0 | 1 | 0 | 0 | 0 | 0 | 0 |
| Neurologic | 0 | 1 | 0 | 0 | 0 | 0 | 0 |
| NeuroVision Imaging | 0 | 0 | 0 | 0 | 0 | 1 | 0 |
| Neurozone MSH | 0 | 1 | 0 | 0 | 0 | 0 | 0 |
| NeuWave Medical | 0 | 0 | 0 | 0 | 2 | 0 | 0 |
| Nihon Kohden | 0 | 1 | 0 | 0 | 0 | 0 | 0 |
| Nines | 0 | 0 | 0 | 0 | 1 | 0 | 0 |
| Nobel Biocare | 0 | 0 | 0 | 0 | 2 | 0 | 0 |
| Nucleus Health | 0 | 0 | 0 | 0 | 1 | 0 | 0 |
| Olea Medical | 0 | 0 | 0 | 0 | 1 | 0 | 0 |
| Oncospace | 0 | 0 | 0 | 0 | 0 | 0 | 0 |
| OnePacs | 0 | 0 | 0 | 0 | 1 | 0 | 0 |
| Optellum | 0 | 0 | 0 | 0 | 0 | 0 | 1 |
| Optima Neuroscience | 0 | 0 | 0 | 0 | 0 | 0 | 0 |
| Optos | 0 | 0 | 0 | 0 | 0 | 1 | 0 |
| Ortho Kinematics | 0 | 0 | 0 | 0 | 2 | 0 | 0 |
| Ortho2 | 0 | 0 | 0 | 0 | 1 | 0 | 0 |
| Ortosoft | 0 | 0 | 0 | 0 | 1 | 0 | 0 |
| Paige | 0 | 0 | 0 | 0 | 0 | 1 | 1 |
| Peek Health | 0 | 0 | 0 | 0 | 1 | 0 | 0 |
| PeraHealth | 2 | 0 | 0 | 0 | 0 | 0 | 0 |
| Perspectum | 0 | 3 | 0 | 0 | 1 | 0 | 0 |
| Philips Medical Systems | 1 | 1 | 0 | 0 | 8 | 0 | 0 |
| Physiq | 0 | 0 | 0 | 0 | 0 | 0 | 0 |
| Pie Medical Imaging | 0 | 0 | 0 | 0 | 3 | 0 | 0 |
| ProKnow Systems | 0 | 0 | 0 | 0 | 1 | 0 | 0 |
| Prowess | 0 | 0 | 0 | 0 | 0 | 0 | 0 |
| PSP | 0 | 0 | 0 | 0 | 1 | 0 | 0 |
| PVmed | 0 | 0 | 0 | 0 | 1 | 0 | 0 |
| QMENTA | 0 | 0 | 0 | 0 | 1 | 0 | 0 |
| Qmetrics Technologies | 0 | 0 | 0 | 0 | 1 | 0 | 0 |
| QUALIFORMED | 0 | 0 | 0 | 0 | 0 | 0 | 0 |
| Quantib | 0 | 0 | 0 | 0 | 4 | 0 | 0 |
| Quantitative Insights | 0 | 0 | 0 | 0 | 0 | 0 | 1 |
| Quantitative Radiology Solutions | 0 | 0 | 0 | 0 | 1 | 0 | 0 |
| Quibim | 0 | 0 | 0 | 0 | 1 | 0 | 0 |
| Quipu srl | 0 | 1 | 0 | 0 | 0 | 0 | 0 |
| Qure AI | 0 | 0 | 0 | 0 | 3 | 0 | 0 |
| Qynapse | 0 | 0 | 0 | 0 | 1 | 0 | 0 |
| Radformation | 0 | 0 | 0 | 0 | 1 | 0 | 0 |
| Radialogica | 0 | 0 | 0 | 0 | 0 | 0 | 0 |
| Radiobotics | 0 | 0 | 0 | 0 | 1 | 0 | 0 |
| RapidAI | 0 | 0 | 0 | 0 | 4 | 0 | 1 |
| Raysearch Laboratories | 0 | 0 | 0 | 0 | 0 | 0 | 0 |
| Resonance Health Ltd | 0 | 3 | 0 | 0 | 0 | 0 | 0 |
| Resonea | 1 | 0 | 0 | 0 | 0 | 0 | 0 |
| Respironics | 0 | 1 | 0 | 0 | 0 | 0 | 0 |
| Samsung Medison | 0 | 0 | 0 | 0 | 3 | 0 | 0 |
| ScreenPoint Medical | 0 | 0 | 0 | 0 | 4 | 0 | 0 |
| Sectra | 0 | 0 | 0 | 0 | 0 | 1 | 0 |
| See-Mode Technologies | 0 | 0 | 0 | 0 | 1 | 0 | 0 |
| Shanghai PZ Medical Technology | 0 | 0 | 0 | 1 | 0 | 0 | 0 |
| SICAT | 0 | 0 | 0 | 0 | 1 | 0 | 0 |
| Siemens | 0 | 0 | 0 | 0 | 44 | 0 | 3 |
| Siris Medical | 0 | 0 | 0 | 0 | 0 | 0 | 0 |
| Sonomed | 0 | 0 | 0 | 0 | 0 | 1 | 0 |
| SOTA Imaging | 0 | 0 | 0 | 0 | 1 | 0 | 0 |
| Sound Options Tinnitus Treatment | 0 | 0 | 1 | 0 | 0 | 0 | 0 |
| Spectrum-Dynamics | 0 | 0 | 0 | 0 | 1 | 0 | 0 |
| Subtle Medical | 0 | 0 | 0 | 0 | 2 | 0 | 0 |
| Surgical Information Sciences | 0 | 0 | 0 | 0 | 2 | 0 | 0 |
| Synaptive Medical | 0 | 0 | 0 | 0 | 1 | 0 | 0 |
| Synopsys | 0 | 0 | 0 | 0 | 1 | 0 | 0 |
| TaiHao Medical | 0 | 0 | 0 | 0 | 3 | 0 | 0 |
| Telerad Tech | 0 | 0 | 0 | 0 | 1 | 0 | 0 |
| Terarecon | 0 | 0 | 0 | 0 | 2 | 0 | 0 |
| TheraPanacea | 0 | 0 | 0 | 0 | 1 | 0 | 0 |
| Therapixel | 0 | 0 | 0 | 0 | 2 | 0 | 0 |
| Therataxis | 0 | 0 | 0 | 0 | 0 | 0 | 0 |
| Therenva | 0 | 0 | 0 | 0 | 2 | 0 | 0 |
| TomTec Imaging Systems | 0 | 0 | 0 | 0 | 3 | 0 | 0 |
| Topcon Healthcare Solutions | 0 | 0 | 0 | 0 | 0 | 4 | 0 |
| Tornier | 0 | 0 | 0 | 0 | 0 | 0 | 0 |
| Toshiba Medical Systems | 0 | 0 | 0 | 0 | 1 | 0 | 0 |
| Trophy | 0 | 0 | 0 | 0 | 0 | 0 | 0 |
| U.S. ARMY DENTAL COMMAND | 0 | 0 | 0 | 0 | 1 | 0 | 0 |
| Ultromics | 0 | 0 | 0 | 0 | 1 | 0 | 1 |
| UMG MEDICAL | 0 | 0 | 0 | 1 | 0 | 0 | 0 |
| United Imaging Healthcare | 0 | 0 | 0 | 0 | 1 | 0 | 0 |
| Varian | 0 | 0 | 0 | 0 | 0 | 0 | 0 |
| Vidan Diagnostics | 0 | 0 | 0 | 0 | 1 | 0 | 0 |
| VidiStar | 0 | 0 | 0 | 0 | 1 | 0 | 0 |
| Virtual Radiologic Corporation | 0 | 0 | 0 | 0 | 1 | 0 | 0 |
| Visage Imaging | 0 | 0 | 0 | 0 | 1 | 0 | 0 |
| Visaris Americas | 0 | 0 | 0 | 0 | 0 | 1 | 0 |
| Visbion | 0 | 0 | 0 | 0 | 1 | 0 | 0 |
| Visible Patient | 0 | 0 | 0 | 0 | 1 | 0 | 0 |
| Visicu | 0 | 0 | 0 | 0 | 0 | 0 | 0 |
| Vista Life Sciences | 0 | 1 | 0 | 0 | 0 | 0 | 0 |
| VISUS Health IT | 0 | 0 | 0 | 0 | 3 | 0 | 0 |
| Visus Technology | 0 | 0 | 0 | 0 | 2 | 0 | 0 |
| Vital Images | 0 | 0 | 0 | 0 | 12 | 0 | 0 |
| VITASYSTEMS | 0 | 1 | 0 | 0 | 0 | 0 | 0 |
| Viz | 0 | 0 | 0 | 0 | 3 | 0 | 0 |
| Vysioneer | 0 | 0 | 0 | 0 | 3 | 0 | 0 |
| Welch Allyn | 0 | 0 | 0 | 0 | 0 | 1 | 0 |
| Wision A.I. | 0 | 0 | 0 | 0 | 0 | 0 | 1 |
| Xiamen Manteia Technology | 0 | 0 | 0 | 0 | 1 | 0 | 0 |
| Zebra Medical Vision | 0 | 0 | 0 | 0 | 8 | 0 | 0 |
| ZEPMED | 0 | 0 | 0 | 0 | 1 | 0 | 0 |
| Ziosoft Inc | 0 | 0 | 0 | 0 | 4 | 0 | 0 |
| ZOLL Medical Corporation | 0 | 0 | 0 | 0 | 0 | 0 | 0 |

Table S7. Contingency table of number of software as a medical device (SaMD) by manufacturers and medical specialty (Item 8-13)

| **Manufacturer Name** | **rehabilitation support** | **remote monitoring** | **review, monitoring and analysis** | **therapy decision support** | **therapy treatment planning** | **visualization, measurement, simulation** |
| --- | --- | --- | --- | --- | --- | --- |
| 20/20 Imaging | 0 | 0 | 0 | 0 | 0 | 0 |
| 3D Industrial Imaging | 0 | 0 | 0 | 0 | 0 | 0 |
| 3D Systems | 0 | 0 | 0 | 0 | 0 | 0 |
| 3Shape | 0 | 0 | 0 | 0 | 0 | 5 |
| 4DX | 0 | 0 | 0 | 0 | 0 | 0 |
| Ablacon | 0 | 0 | 0 | 0 | 0 | 0 |
| Acuitas Medical | 0 | 0 | 0 | 0 | 0 | 0 |
| ADAS3D Medical | 0 | 0 | 0 | 0 | 0 | 0 |
| ADM Diagnostics | 0 | 0 | 0 | 0 | 0 | 0 |
| Advanced Brain Monitoring | 0 | 0 | 0 | 0 | 0 | 0 |
| Advantis Medical Imaging | 0 | 0 | 0 | 0 | 0 | 0 |
| Agfa HealthCare | 0 | 0 | 0 | 0 | 0 | 0 |
| AI Metrics | 0 | 0 | 0 | 0 | 0 | 0 |
| AIbolit Technologies | 0 | 0 | 0 | 0 | 0 | 0 |
| Aidoc | 0 | 0 | 0 | 0 | 0 | 0 |
| AIRS Medical | 0 | 0 | 0 | 0 | 0 | 0 |
| Airstrip Technologies | 0 | 0 | 0 | 0 | 0 | 0 |
| AIT Austrian Institute Of Technology | 0 | 0 | 1 | 0 | 0 | 0 |
| Al Pacheco | 0 | 0 | 0 | 1 | 0 | 0 |
| AlertWatch | 0 | 0 | 0 | 0 | 0 | 0 |
| Algomedica | 0 | 0 | 0 | 0 | 0 | 0 |
| AliveCor | 0 | 0 | 0 | 0 | 0 | 0 |
| Alphatec Spine | 0 | 0 | 0 | 0 | 0 | 0 |
| AmCad BioMed | 0 | 0 | 0 | 0 | 0 | 0 |
| Anatomage Inc. | 0 | 0 | 0 | 0 | 0 | 0 |
| Apollo Medical Imaging Technology | 0 | 0 | 0 | 0 | 0 | 0 |
| Apple | 0 | 2 | 0 | 0 | 0 | 0 |
| Apteryx Imaging | 0 | 0 | 0 | 0 | 0 | 0 |
| ArchForm | 0 | 0 | 0 | 0 | 0 | 1 |
| ArteryFlow | 0 | 0 | 0 | 0 | 0 | 0 |
| Arterys | 0 | 0 | 0 | 0 | 0 | 0 |
| ArthroMeda | 0 | 0 | 0 | 0 | 0 | 0 |
| ASCEND HiT | 0 | 0 | 0 | 0 | 0 | 0 |
| Austrian Institute of Technology | 0 | 0 | 1 | 0 | 0 | 0 |
| Avicenna.ai | 0 | 0 | 0 | 0 | 0 | 0 |
| Behold.ai | 0 | 0 | 0 | 0 | 0 | 0 |
| Best NOMOS | 0 | 0 | 0 | 0 | 1 | 0 |
| Biobot Surgical Pte Ltd | 0 | 0 | 0 | 0 | 0 | 0 |
| Biofourmis | 0 | 1 | 0 | 0 | 0 | 0 |
| Biolight | 0 | 0 | 0 | 0 | 0 | 0 |
| BioMet | 0 | 0 | 0 | 0 | 0 | 0 |
| BoxView | 0 | 1 | 0 | 0 | 0 | 0 |
| BrainLAB | 0 | 0 | 0 | 0 | 2 | 0 |
| BrainMaster Technologies | 0 | 0 | 0 | 0 | 0 | 0 |
| BrainNow | 0 | 0 | 0 | 0 | 0 | 0 |
| Brainomix | 0 | 0 | 0 | 0 | 0 | 0 |
| Broncus Technologies | 0 | 0 | 0 | 0 | 0 | 0 |
| B-Secur | 0 | 1 | 0 | 0 | 0 | 0 |
| Cadwell Laboratories | 0 | 0 | 0 | 0 | 0 | 0 |
| Calgary Scientific | 0 | 0 | 0 | 0 | 0 | 0 |
| Canon Medical Systems | 0 | 0 | 0 | 0 | 0 | 0 |
| Caption Health | 0 | 0 | 0 | 0 | 0 | 0 |
| Cardiologs | 0 | 1 | 0 | 0 | 0 | 0 |
| Carefusion | 0 | 0 | 0 | 0 | 0 | 0 |
| Carestream | 0 | 0 | 0 | 0 | 0 | 0 |
| Caristo Diagnostics | 0 | 0 | 0 | 0 | 0 | 0 |
| Carl Zeiss Meditec | 0 | 0 | 0 | 0 | 0 | 0 |
| CAScination | 0 | 0 | 0 | 0 | 0 | 0 |
| CathWorks | 0 | 0 | 0 | 0 | 0 | 2 |
| CDB Corp | 0 | 0 | 0 | 0 | 0 | 1 |
| Ceevra, Inc. | 0 | 0 | 0 | 0 | 0 | 0 |
| Change Healthcare | 0 | 0 | 0 | 0 | 0 | 0 |
| Circle Cardiovascular Imaging | 0 | 0 | 0 | 0 | 0 | 0 |
| ClariPi | 0 | 0 | 0 | 0 | 0 | 0 |
| Claritas HealthTech | 0 | 0 | 0 | 0 | 0 | 0 |
| ClearView Diagnostics | 0 | 0 | 0 | 0 | 0 | 0 |
| Cleerly | 0 | 0 | 0 | 0 | 0 | 0 |
| CLEW Medical | 0 | 0 | 0 | 1 | 0 | 0 |
| Client Outlook | 0 | 0 | 0 | 0 | 0 | 0 |
| CONSOLIDATED RESEARCH | 0 | 0 | 0 | 0 | 0 | 0 |
| Cordiana Medical Informatics | 0 | 0 | 0 | 0 | 0 | 0 |
| CORELINESOFT | 0 | 0 | 0 | 0 | 0 | 0 |
| CorTechs Labs | 0 | 0 | 0 | 0 | 0 | 0 |
| CorticoMetrics | 0 | 0 | 0 | 0 | 0 | 0 |
| Covidien | 0 | 0 | 0 | 0 | 0 | 0 |
| Cuptimize | 0 | 0 | 0 | 0 | 0 | 0 |
| CureMetrix | 0 | 0 | 0 | 0 | 0 | 0 |
| Curve Beam | 0 | 0 | 0 | 0 | 0 | 0 |
| Cycle Clarity | 0 | 0 | 0 | 0 | 0 | 0 |
| Cydar Medical | 0 | 0 | 0 | 0 | 0 | 0 |
| de Gotzen | 0 | 0 | 0 | 0 | 0 | 0 |
| Decisio Health | 0 | 0 | 0 | 0 | 0 | 0 |
| Deep01 Technology | 0 | 0 | 0 | 0 | 0 | 0 |
| DeepHealth | 0 | 0 | 0 | 0 | 0 | 0 |
| DeepLook | 0 | 0 | 0 | 0 | 0 | 0 |
| Deepvoxel | 0 | 0 | 0 | 0 | 0 | 0 |
| Densitas | 0 | 0 | 0 | 0 | 0 | 0 |
| Dentsply Sirona | 0 | 0 | 0 | 0 | 0 | 1 |
| Dexin Medical Imaging Technology | 0 | 0 | 0 | 0 | 0 | 0 |
| Digital Core Technology | 0 | 0 | 0 | 0 | 0 | 0 |
| DOSIsoft | 0 | 0 | 0 | 0 | 1 | 0 |
| DreaMed Diabetes | 0 | 0 | 0 | 2 | 0 | 0 |
| DRGEM | 0 | 0 | 0 | 0 | 0 | 0 |
| Dürr Dental | 0 | 0 | 0 | 0 | 0 | 0 |
| EB Neuro | 0 | 0 | 0 | 0 | 0 | 0 |
| EBM Technologies | 0 | 0 | 0 | 0 | 0 | 0 |
| EchoPixel | 0 | 0 | 0 | 0 | 0 | 0 |
| Eckert & Ziegler Bebig SA | 0 | 0 | 0 | 0 | 1 | 0 |
| Edan Instruments | 0 | 0 | 0 | 0 | 0 | 0 |
| EDDA Technology | 0 | 0 | 0 | 0 | 0 | 0 |
| eko.ai | 0 | 0 | 0 | 0 | 0 | 0 |
| Elekta | 0 | 0 | 0 | 0 | 4 | 0 |
| Eli Lilly | 0 | 0 | 0 | 1 | 0 | 0 |
| ElMindA | 0 | 0 | 0 | 0 | 0 | 0 |
| Elucid | 0 | 0 | 0 | 0 | 0 | 0 |
| EndoVantage | 0 | 0 | 0 | 0 | 0 | 1 |
| EnsoData | 0 | 0 | 0 | 0 | 0 | 0 |
| Epilog | 0 | 0 | 0 | 0 | 0 | 0 |
| Epiphany Healthcare | 0 | 0 | 0 | 0 | 0 | 0 |
| Etiometry | 0 | 1 | 0 | 0 | 0 | 0 |
| Ever Fortune.AI | 0 | 0 | 0 | 0 | 0 | 0 |
| eVolve PACS | 0 | 0 | 0 | 0 | 0 | 0 |
| Ewoosoft | 0 | 0 | 0 | 0 | 0 | 0 |
| EXINI Diagnostics | 0 | 0 | 0 | 0 | 0 | 0 |
| exocad GmbH | 0 | 0 | 0 | 0 | 0 | 0 |
| FEops | 0 | 0 | 0 | 0 | 0 | 1 |
| Fifth Eye | 0 | 0 | 0 | 1 | 0 | 0 |
| Firstsource | 0 | 0 | 0 | 0 | 0 | 0 |
| Fitbit | 0 | 1 | 0 | 0 | 0 | 0 |
| FLUIDDA | 0 | 0 | 0 | 0 | 0 | 0 |
| Fujifilm Medical Systems | 0 | 0 | 0 | 0 | 0 | 0 |
| Galgo Medical | 0 | 0 | 0 | 0 | 0 | 0 |
| GE Healthcare | 0 | 0 | 0 | 0 | 1 | 0 |
| Genesis Software Innovations | 0 | 0 | 0 | 0 | 0 | 0 |
| GMV | 0 | 0 | 0 | 0 | 1 | 0 |
| Haag-Streit USA | 0 | 0 | 0 | 0 | 0 | 0 |
| Halifax Biomedical | 0 | 0 | 0 | 0 | 0 | 0 |
| HDX WILL | 0 | 0 | 0 | 0 | 0 | 0 |
| HealthMyne | 0 | 0 | 0 | 0 | 0 | 0 |
| Heart Imaging Technologies | 0 | 0 | 0 | 0 | 0 | 0 |
| HeartFlow | 0 | 0 | 0 | 0 | 0 | 6 |
| Highmark Interactive | 0 | 0 | 0 | 0 | 0 | 0 |
| Hinacom | 0 | 0 | 0 | 0 | 0 | 0 |
| Hologic | 0 | 0 | 0 | 0 | 0 | 0 |
| iCAD | 0 | 0 | 0 | 0 | 0 | 0 |
| icometrix | 0 | 0 | 0 | 0 | 0 | 0 |
| ImageBiopsy Lab | 0 | 0 | 0 | 0 | 0 | 0 |
| Imagen Technologies | 0 | 0 | 0 | 0 | 0 | 0 |
| Imaging Biometrics | 0 | 0 | 0 | 0 | 0 | 0 |
| Imaging Sciences International | 0 | 0 | 0 | 0 | 0 | 0 |
| Imbio | 0 | 0 | 0 | 0 | 0 | 0 |
| IMPAC Medical System | 0 | 0 | 0 | 0 | 1 | 0 |
| Infervision | 0 | 0 | 0 | 0 | 0 | 0 |
| Infinitt | 0 | 0 | 0 | 0 | 0 | 0 |
| Intelligent Retinal Imaging Systems | 0 | 0 | 0 | 0 | 0 | 0 |
| Intellijoint Surgical | 0 | 0 | 0 | 0 | 0 | 0 |
| International Medical Solutions | 0 | 0 | 0 | 0 | 0 | 0 |
| Intoware | 0 | 0 | 0 | 0 | 0 | 0 |
| Intuitive Surgical | 0 | 0 | 0 | 0 | 0 | 0 |
| InVivo Therapeutics | 0 | 0 | 0 | 0 | 0 | 0 |
| Jintronix | 1 | 0 | 0 | 0 | 0 | 0 |
| JointVue | 0 | 0 | 0 | 0 | 0 | 0 |
| JPI Healthcare Solutions | 0 | 0 | 0 | 0 | 0 | 0 |
| KEYA MEDICAL | 0 | 0 | 0 | 0 | 0 | 0 |
| Koios Medical | 0 | 0 | 0 | 0 | 0 | 0 |
| LEAD Technologies, Inc. | 0 | 0 | 0 | 0 | 0 | 0 |
| Lexi | 0 | 0 | 0 | 0 | 0 | 0 |
| Limbus AI | 0 | 0 | 0 | 0 | 0 | 0 |
| LK Consulting | 0 | 0 | 0 | 0 | 0 | 0 |
| Luminopia | 0 | 0 | 0 | 0 | 0 | 0 |
| Lunit | 0 | 0 | 0 | 0 | 0 | 0 |
| Materialise | 0 | 0 | 0 | 0 | 0 | 1 |
| MATH RESOLUTIONS | 0 | 0 | 0 | 0 | 1 | 0 |
| MaxQ-AI | 0 | 0 | 0 | 0 | 0 | 0 |
| McKesson | 0 | 0 | 0 | 0 | 0 | 0 |
| Medic Vision | 0 | 0 | 0 | 0 | 0 | 0 |
| MedicalCommunications | 0 | 0 | 0 | 0 | 0 | 0 |
| Medicrea | 0 | 0 | 0 | 0 | 0 | 0 |
| Medis Medical Imaging Systems BV | 0 | 0 | 0 | 0 | 0 | 0 |
| MEDIVIS | 0 | 0 | 0 | 0 | 0 | 0 |
| Medmind | 0 | 0 | 0 | 0 | 0 | 0 |
| Medo.ai | 0 | 0 | 0 | 0 | 0 | 0 |
| Medsynaptic | 0 | 0 | 0 | 0 | 0 | 0 |
| Medusa Medical Technologies | 0 | 0 | 0 | 0 | 0 | 0 |
| Medviso | 0 | 0 | 0 | 0 | 0 | 0 |
| Merge | 0 | 0 | 0 | 0 | 0 | 0 |
| metaMe Health | 0 | 0 | 0 | 0 | 0 | 0 |
| MetriTrack | 0 | 0 | 0 | 0 | 0 | 0 |
| MeVis Medical Solutions AG | 0 | 0 | 0 | 0 | 0 | 0 |
| Microsoft | 0 | 0 | 0 | 0 | 0 | 0 |
| Mint Medical | 0 | 0 | 0 | 0 | 0 | 0 |
| Mirada Medical | 0 | 0 | 0 | 0 | 0 | 0 |
| Mobius Medical Systems | 0 | 0 | 0 | 0 | 3 | 0 |
| Monarch Medical Technologies | 0 | 0 | 0 | 3 | 0 | 0 |
| Motilent | 0 | 0 | 0 | 0 | 0 | 0 |
| MRIguidance | 0 | 0 | 0 | 0 | 0 | 0 |
| MultiModal Health | 0 | 0 | 0 | 0 | 0 | 0 |
| Myocardial Solutions | 0 | 0 | 0 | 0 | 0 | 0 |
| Natural Cycles | 0 | 1 | 0 | 0 | 0 | 0 |
| Natus Medical | 0 | 0 | 0 | 0 | 0 | 0 |
| NEOLOGICA | 0 | 0 | 0 | 0 | 0 | 0 |
| NeuroChaos Solutions | 0 | 0 | 0 | 0 | 0 | 0 |
| Neurologic | 0 | 0 | 0 | 0 | 0 | 0 |
| NeuroVision Imaging | 0 | 0 | 0 | 0 | 0 | 0 |
| Neurozone MSH | 0 | 0 | 0 | 0 | 0 | 0 |
| NeuWave Medical | 0 | 0 | 0 | 0 | 0 | 0 |
| Nihon Kohden | 0 | 0 | 0 | 0 | 0 | 0 |
| Nines | 0 | 0 | 0 | 0 | 0 | 0 |
| Nobel Biocare | 0 | 0 | 0 | 0 | 0 | 1 |
| Nucleus Health | 0 | 0 | 0 | 0 | 0 | 0 |
| Olea Medical | 0 | 0 | 0 | 0 | 0 | 0 |
| Oncospace | 0 | 0 | 0 | 0 | 1 | 0 |
| OnePacs | 0 | 0 | 0 | 0 | 0 | 0 |
| Optellum | 0 | 0 | 0 | 0 | 0 | 0 |
| Optima Neuroscience | 0 | 0 | 1 | 0 | 0 | 0 |
| Optos | 0 | 0 | 0 | 0 | 0 | 0 |
| Ortho Kinematics | 0 | 0 | 0 | 0 | 0 | 0 |
| Ortho2 | 0 | 0 | 0 | 0 | 0 | 0 |
| Ortosoft | 0 | 0 | 0 | 0 | 0 | 0 |
| Paige | 0 | 0 | 0 | 0 | 0 | 0 |
| Peek Health | 0 | 0 | 0 | 0 | 0 | 0 |
| PeraHealth | 0 | 0 | 0 | 0 | 0 | 0 |
| Perspectum | 0 | 0 | 0 | 0 | 0 | 0 |
| Philips Medical Systems | 0 | 1 | 0 | 0 | 1 | 0 |
| Physiq | 0 | 4 | 0 | 0 | 0 | 0 |
| Pie Medical Imaging | 0 | 0 | 0 | 0 | 0 | 0 |
| ProKnow Systems | 0 | 0 | 0 | 0 | 0 | 0 |
| Prowess | 0 | 0 | 0 | 0 | 1 | 0 |
| PSP | 0 | 0 | 0 | 0 | 0 | 0 |
| PVmed | 0 | 0 | 0 | 0 | 0 | 0 |
| QMENTA | 0 | 0 | 0 | 0 | 0 | 0 |
| Qmetrics Technologies | 0 | 0 | 0 | 0 | 0 | 0 |
| QUALIFORMED | 0 | 0 | 0 | 0 | 1 | 0 |
| Quantib | 0 | 0 | 0 | 0 | 0 | 0 |
| Quantitative Insights | 0 | 0 | 0 | 0 | 0 | 0 |
| Quantitative Radiology Solutions | 0 | 0 | 0 | 0 | 0 | 0 |
| Quibim | 0 | 0 | 0 | 0 | 0 | 0 |
| Quipu srl | 0 | 0 | 0 | 0 | 0 | 0 |
| Qure AI | 0 | 0 | 0 | 0 | 0 | 0 |
| Qynapse | 0 | 0 | 0 | 0 | 0 | 0 |
| Radformation | 0 | 0 | 0 | 0 | 1 | 0 |
| Radialogica | 0 | 0 | 0 | 0 | 1 | 0 |
| Radiobotics | 0 | 0 | 0 | 0 | 0 | 0 |
| RapidAI | 0 | 0 | 0 | 0 | 0 | 0 |
| Raysearch Laboratories | 0 | 0 | 0 | 0 | 3 | 0 |
| Resonance Health Ltd | 0 | 0 | 0 | 0 | 0 | 0 |
| Resonea | 0 | 0 | 0 | 0 | 0 | 0 |
| Respironics | 0 | 0 | 0 | 0 | 0 | 0 |
| Samsung Medison | 0 | 0 | 0 | 0 | 0 | 0 |
| ScreenPoint Medical | 0 | 0 | 0 | 0 | 0 | 0 |
| Sectra | 0 | 0 | 0 | 0 | 0 | 0 |
| See-Mode Technologies | 0 | 0 | 0 | 0 | 0 | 0 |
| Shanghai PZ Medical Technology | 0 | 0 | 0 | 0 | 0 | 0 |
| SICAT | 0 | 0 | 0 | 0 | 0 | 0 |
| Siemens | 0 | 0 | 0 | 0 | 1 | 0 |
| Siris Medical | 0 | 0 | 0 | 0 | 1 | 0 |
| Sonomed | 0 | 0 | 0 | 0 | 0 | 0 |
| SOTA Imaging | 0 | 0 | 0 | 0 | 0 | 0 |
| Sound Options Tinnitus Treatment | 0 | 0 | 0 | 0 | 0 | 0 |
| Spectrum-Dynamics | 0 | 0 | 0 | 0 | 0 | 0 |
| Subtle Medical | 0 | 0 | 0 | 0 | 0 | 0 |
| Surgical Information Sciences | 0 | 0 | 0 | 0 | 0 | 0 |
| Synaptive Medical | 0 | 0 | 0 | 0 | 0 | 0 |
| Synopsys | 0 | 0 | 0 | 0 | 0 | 0 |
| TaiHao Medical | 0 | 0 | 0 | 0 | 0 | 0 |
| Telerad Tech | 0 | 0 | 0 | 0 | 0 | 0 |
| Terarecon | 0 | 0 | 0 | 0 | 0 | 0 |
| TheraPanacea | 0 | 0 | 0 | 0 | 0 | 0 |
| Therapixel | 0 | 0 | 0 | 0 | 0 | 0 |
| Therataxis | 0 | 0 | 0 | 0 | 1 | 0 |
| Therenva | 0 | 0 | 0 | 0 | 0 | 0 |
| TomTec Imaging Systems | 0 | 0 | 0 | 0 | 0 | 0 |
| Topcon Healthcare Solutions | 0 | 0 | 0 | 0 | 0 | 0 |
| Tornier | 0 | 0 | 0 | 0 | 0 | 1 |
| Toshiba Medical Systems | 0 | 0 | 0 | 0 | 0 | 0 |
| Trophy | 0 | 0 | 0 | 0 | 0 | 1 |
| U.S. ARMY DENTAL COMMAND | 0 | 0 | 0 | 0 | 0 | 0 |
| Ultromics | 0 | 0 | 0 | 0 | 0 | 0 |
| UMG MEDICAL | 0 | 0 | 0 | 0 | 0 | 0 |
| United Imaging Healthcare | 0 | 0 | 0 | 0 | 0 | 0 |
| Varian | 0 | 0 | 0 | 0 | 16 | 0 |
| Vidan Diagnostics | 0 | 0 | 0 | 0 | 0 | 0 |
| VidiStar | 0 | 0 | 0 | 0 | 0 | 0 |
| Virtual Radiologic Corporation | 0 | 0 | 0 | 0 | 0 | 0 |
| Visage Imaging | 0 | 0 | 0 | 0 | 0 | 0 |
| Visaris Americas | 0 | 0 | 0 | 0 | 0 | 0 |
| Visbion | 0 | 0 | 0 | 0 | 0 | 0 |
| Visible Patient | 0 | 0 | 0 | 0 | 0 | 0 |
| Visicu | 0 | 3 | 0 | 0 | 0 | 0 |
| Vista Life Sciences | 0 | 0 | 0 | 0 | 0 | 0 |
| VISUS Health IT | 0 | 0 | 0 | 0 | 0 | 0 |
| Visus Technology | 0 | 0 | 0 | 0 | 0 | 0 |
| Vital Images | 0 | 0 | 0 | 0 | 0 | 0 |
| VITASYSTEMS | 0 | 0 | 0 | 0 | 0 | 0 |
| Viz | 0 | 0 | 0 | 0 | 0 | 0 |
| Vysioneer | 0 | 0 | 0 | 0 | 0 | 0 |
| Welch Allyn | 0 | 0 | 0 | 0 | 0 | 0 |
| Wision A.I. | 0 | 0 | 0 | 0 | 0 | 0 |
| Xiamen Manteia Technology | 0 | 0 | 0 | 0 | 0 | 0 |
| Zebra Medical Vision | 0 | 0 | 0 | 0 | 0 | 0 |
| ZEPMED | 0 | 0 | 0 | 0 | 0 | 0 |
| Ziosoft Inc | 0 | 0 | 0 | 0 | 0 | 0 |
| ZOLL Medical Corporation | 0 | 1 | 0 | 0 | 0 | 0 |
