# Supplementary figures and images for: Innovation Process and Industrial System of US Food and Drug Administration–Approved Software as a Medical Device: Review and Content Analysis
Source: J Med Internet Res. 2023 Nov 24;25:e47505. doi: 10.2196/47505 (PMC10709785; doi:10.2196/47505)

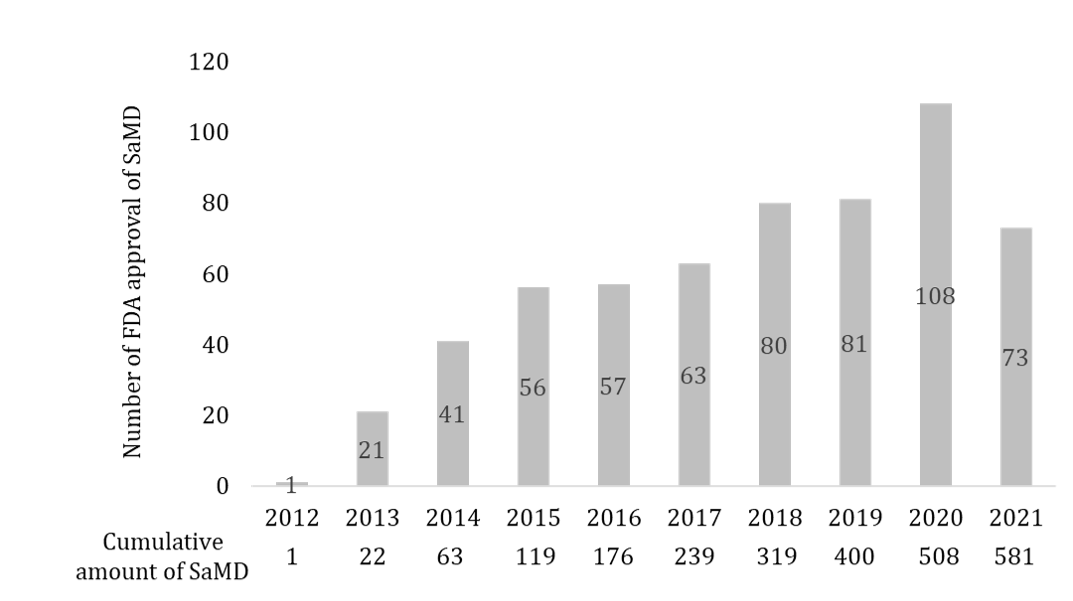

Supplement: Multimedia Appendix 2 [file jmir_v25i1e47505_app2.png]
